# Next generation smart functionalized nanocomposites for ultrasensitive and real time detection of purine derived metabolic biomarkers

**DOI:** 10.1039/d6ra04619c

**Published:** 2026-09-25

**Authors:** Rooh Ullah, Jamshed Ali, Saif Ullah, Rasha M. K. Mohamed, Afzal Shah, Mustafa Tuzen

**Affiliations:** a Tokat Gaziosmanpasa University, Faculty of Science and Arts, Chemistry Department Tokat 60250 Turkey roohullah@uot.edu.pk mustafa.tuzen@gop.edu.tr; b Department of Chemistry, University of Turbat Balochistan Pakistan; c School of Agricultural Engineering and Food Science, Shandong University of Technology, No. 266 Xincun Xilu Zibo Shandong 255049 China; d Department of Chemistry, College of Science, Jouf University Sakaka Aljouf 72341 Saudi Arabia; e Department of Chemistry, Faculty of Science, Assiut University Assiut 71516 Egypt; f Department of Chemistry, Quaid-i-Azam University Islamabad 45320 Pakistan afzals_qau@yahoo.com

## Abstract

The monitoring of metabolic biomarkers is currently undergoing a radical change from traditional centralized laboratory standards to decentralized point-of-care testing, particularly in the management of metabolic disorders, such as gout, and renal dysfunction. This review explores next-generation functionalized nanocomposites such as metal–organic frameworks, two-dimensional (2D) transition-metal carbides, nitrides, and carbonitrides (MXenes) and Single-Atom Nanozymes (SANs) which function as sensitive elements in sensing platforms for biomarkers detection and serve as robust, non-enzymatic mimics to address the stability challenges associated with natural enzymes. Hydrothermal and green co-precipitation methods are strategic routes to fabrication, with enhanced scalability and catalytic activity. Representative analytical benchmarks demonstrate the superior performance of these platforms for metabolic biomarkers detection achieving a picomolar limit of detection (LOD) of 102 pM for Ag-Fe_2_O_3_@PAn composites and an ultra-trace fluorescence LOD of 15 pM for tetrakis(4-carboxyphenyl)porphyrin (TCPP)-Zn_0.1_Co_2_O_4_ nanorods. In addition, a Nitrogen-coordinated Cobalt Single-Atom/Graphene-based Nanozyme (A–Co–NG) exhibits an extensively linear range of up to 41 950 µM with a 33 µM LOD. The review emphasizes novel biomimetic engineering approaches designed to address ongoing real-world issues, including biofouling and the interference caused by the overlapping signals from ascorbic acid and dopamine. The ingenuity and adaptability of these materials in wearable formats and applications, such as smart diapers and smartphone-assisted colorimetric sensors, offers a non-invasive roadmap for continuous real-time monitoring of metabolic processes and personalized health care. This review offers a comprehensive survey of the technological landscape for metabolic biomarkers measurements, transitioning from centralized laboratory diagnostics to decentralized point-of-care testing (POCT). It presents sophisticated sensing systems that employ highly sensitive sensors for the instantaneous identification of purine-based metabolic biomarkers, indicating a notable shift in technological advancements and their implications for human interaction. Furthermore, the review examines significant obstacles that impact the effectiveness of these technologies, limiting their practical use in real-world situations.

## Abstract 2

The monitoring of metabolic biomarkers is currently undergoing a radical change from traditional centralized laboratory standards to decentralized point-of-care testing, particularly in the management of metabolic disorders.

## Graphical abstract

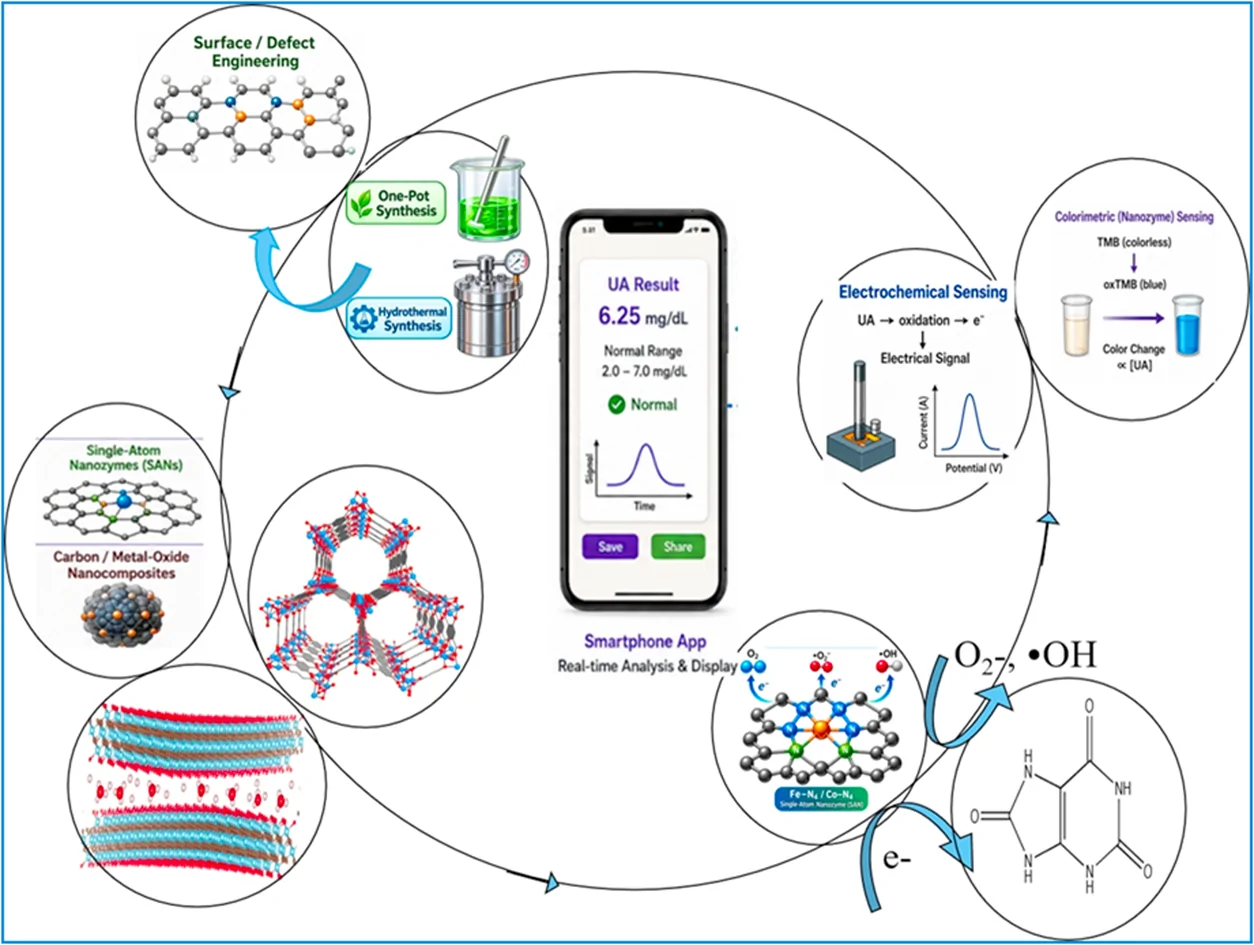

## 1. Introduction

Purines are critical cellular components with many functions, including the regulation of energy metabolism and facilitation of signal transduction. Their vital roles encompass key physiological processes in platelets, muscle tissues, and neurotransmission. In humans, uric acid (UA) is the final product of purine metabolism, making its measurement crucial for diagnostics and health evaluations. High concentrations of UA in blood (hyperuricemia) are a key diagnostic indicator for gout, an inflammatory arthritis affecting millions globally. Moreover, elevated UA levels are linked to several health problems, including cardiovascular disease, metabolic syndrome, and kidney conditions, such as stone formation. Additionally, changes in UA levels are associated with certain neurological disorders, such as multiple sclerosis and Parkinson's disease, highlighting the importance of UA in various medical conditions.^1^ The need for new techniques for UA detection arises from the limitations of conventional laboratory techniques. High-performance liquid chromatography (HPLC) and enzymatic assays can accurately quantify UA, but their complexity, high costs, and the need for specialized personnel and sophisticated equipment limit their applicability for point-of-care (POC) monitoring. In contrast, there is a growing demand for low-cost and distributed point-of-care testing (POCT) that can be more readily adopted in diverse healthcare settings. Integration with smartphone technologies provides remote sample analysis, data processing and tele-health monitoring, enabling a transition from point-of-care diagnostics to smart medical care. However, it is challenging to develop portable UA sensors. This is because of interference from other species in biological fluids.^2^ Substances such as ascorbic acid (AA) and dopamine (DA) have similar oxidation potential as UA, leading to interference and detection errors. This situation underscores the urgent need for the development of sensitive and UA specific sensors. The use of functional nanomaterials and microfluidic paper-based analytical devices (µPADs) are among the new developments for improved efficiency and mobility. However, integration, reproducibility and stability to create effective diagnostic and personalized medicine tools are key challenges to be overcome.

The evolution of wearable technology and smartphone-enabled point-of-care testing is seen as a shift from natural enzymes to effective nanocomposites and single-atom nanozymes (SANs). This shift is also associated with the emergence of one-pot and eco-friendly synthesis techniques. Functional nanocomposites and SANs offer improved catalytic functions, adjustable active sites, and greater stability, addressing the drawbacks linked to natural enzymes. The use of one-pot and green synthesis methods enables the production of these materials in a simpler, environmentally friendly, and possibly scalable way.^3^ Improvements in material design and manufacturing techniques allow for the incorporation of these innovations into portable, flexible, and wearable sensing devices, facilitating quick and real-time biomarker identification. As a result, this review is structured around the core theme of “materials-fabrication-translation”, highlighting the advancement of functional sensing materials and their transformation into sustainable manufacturing approaches, ultimately aiding in the development of effective, decentralized, and intelligent diagnostic systems.^4^

The current method of diagnosing UA and other biomarkers, including glucose and antibiotics, primarily rely on lab techniques. HPLC, gas chromatography mass spectrometry (GC-MS), and enzymatic methods are reference standards that have high sensitivity and accuracy. However, they are expensive, involve complicated machinery and demand highly trained personnel to operate effectively. They cannot be carried out and fixed installations must be installed in laboratories. Moreover, the efforts of sample preparation can be labor-intensive, and carries the risk of contamination or loss of samples. The main technical problems of the current systems are selectivity and sensitivity. The UA detection by electrochemical sensing technique is further complicated by the presence of co-existing species such as AA and DA with overlapping oxidation potentials.^2,5^ This complicates the detection of UA by voltammetric techniques using conventional electrodes. Although enzymatic biosensors are very specific, they are expensive to manufacture, have a low shelf life and are sensitive to environmental conditions such as temperature, pH levels and humidity. These factors can lead to a loss of enzyme activity and sensor performance. Amplification and sensitivity issues are also common, especially in miniature and/or porous materials used in novel sensing systems. Low detection limits and broad dynamic ranges are challenging to achieve, particularly for UA detection in biological samples. This limits the effectiveness of many current systems for clinical applications.^6,7^

The move towards POCT devices, such as µPADs and integrated sensors compatible with smartphones introduces further challenges. While µPADs are attractive due to their cost-effectiveness and portability, they usually suffer from stability issues with the reagents and environmental factors such as humidity, microorganisms, *etc.* These factors can affect their storage and overall stability.^8,9^ Similarly, optical biosensing technologies, which use smartphones, are influenced by changes in light conditions and the characteristics of the smartphone camera. This can influence the signal detection and reproducibility of the results, particularly outdoors. Besides technical issues, there are also challenges in commercializing UA sensing technologies.^10^ Lack of standardized protocols for manufacturing, calibrating and analyzing data can affect device reproducibility. Furthermore, the regulatory landscape for commercialization can be challenging and only technologies that can be used in diagnostics can meet the stringent requirements of safety and performance standards. Moreover, ensuring security and privacy are extremely essential to smartphone sensing technologies, given the personal nature of health data. To address these challenges, a cross-disciplinary approach is required, and innovations in materials, sensors and data analytics are needed. Improving stability, selectivity and affordability of the technology, as well as ensuring compliance and data security, are essential for the technology to be widely adopted.^11,12^

This review provides a unified picture of the development of next generation sensing platforms for metabolic biomarkers from the purine family, specifically uric acid, through the perspectives of the material evolution, sustainable manufacturing, and point-of-care translation. The core idea is that these developments should not be viewed in isolation; rather, they signify a continuum of shifts from conventional laboratory analyses, which depend on enzyme activity, to reliable, decentralized, and smart diagnostic systems. First, functional nanocomposites, metal–organic frameworks (MOFs), two-dimensional transition-metal carbides, nitrides, and carbonitrides (MXenes), and single-atom nanozymes offer high catalytic activity, adjustable catalytic sites, stability and selectivity (Table 1), which are superior to the conventional sensing interfaces. Second, the synthesis approaches for these materials involve a single-step and green process can provide structures and catalytic properties with a controlled morphology and scale. Overall, the integration of these high-tech sensing materials into the platforms of microfluidic paper analytical devices (µPADs), wearable sensors and POC test platforms that are smartphone-based could allow for rapid and non-invasive and even continuous monitoring of biomarkers. Thus, this review adheres to the materials-fabrication-translation pathway to rigorously examine the conversion of proposed innovations in nanocomposite design and sustainable synthesis into clinically applicable sensing technologies, while also emphasizing potential challenges related to selectivity, biofouling, reproducibility, scalability, biosafety, standardization, and digitally integrated sensing devices.

**Table 1:** General comparison, analytical characteristics, and challenges of metabolic biomarker detection methods^a^

Method/technology	Detection time/throughput	Economic factors (cost)	Technical challenges & limitations	Ref.
RGO–ZnO composite electrode	Rapid voltammetric response	Low cost; uses spontaneous one-pot green synthesis at room temperature	Requires resolving overlapping oxidation potentials of ascorbic acid (AA) and dopamine (DA)	13
Smartphone colorimetric (AgTNPs)	45 min incubation at 45 °C	Cost-effective; eliminates need for intricate lab spectrophotometers	Highly susceptible to ambient light variability and hardware disparities between smartphone sensors	14
µPADs (paper-based devices)	Rapid results, often within minutes	Extremely low fabrication cost; simple, scalable, and disposable	Unstable reagents and sensitivity to environmental factors like moisture and microbial contamination	6
Medical smartphone analyzer	Real-time, dynamic monitoring	Device cost 60; employs disposable enzymatic test strips	Accuracy and reliability often do not match professional, dedicated lab equipment	15
Dual-FRET aptasensor	10 min rapid detection	Uses 3D-printed portable device for personalized monitoring	High signal background; requires careful calibration against serum matrix interferences	1 and 6
Traditional lab methods (HPLC/GC-MS)	Time-intensive due to complex pretreatment	High analysis cost; requires specialized personnel and expensive fixed equipment	Bulky, non-portable, and unsuitable for frequent decentralized or at-home testing	16

a Silver triangular nanoplates/silver nanoplates (AgTNPs), Förster resonance energy transfer (FRET).

## 2. Principles of uric acid detection

**Fig. 1:**
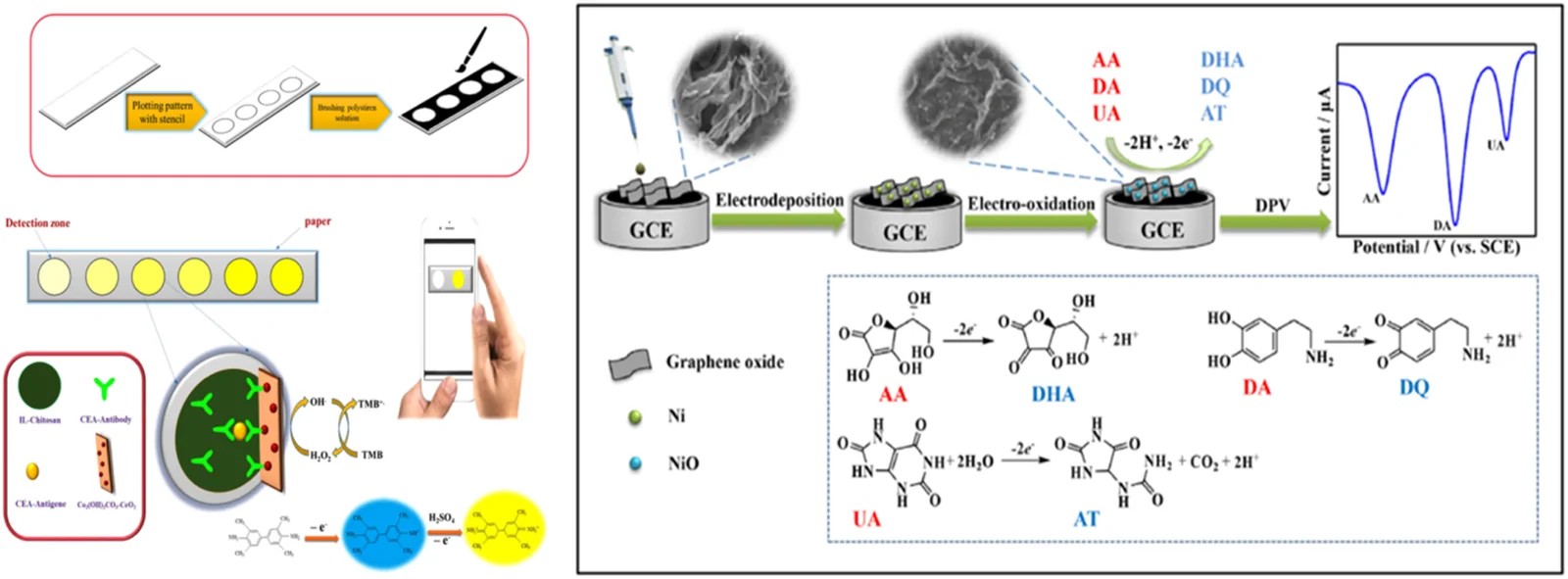
Schematic illustration of NiO/graphene oxide-modified electrode fabrication and its electrochemical mechanism for simultaneous DPV detection of uric acid and its signals discrimination from DA and AA interferents. This figure has been reproduced from ref. 22 with permission from ELSEVIER, copyright 2019.

The detection of uric acid in biological fluids is primarily based on the principles of electrochemical, optical and biomimetic methods. Electrochemical sensors are voltammetric based, *e.g.*, cyclic voltammetry (CV) and differential pulse voltammetry (DPV), which generate a current as the acid is oxidized at the electrode surface.^17^ As shown in the Fig. 1, electrodes are functionalized by nanomaterials such as graphene, MXene and nickel oxide (NiO) that enhance the catalytic activity, give them a high surface area and help in separation of the oxidation peak of UA from the signals of interferents such as ascorbic acid (AA) and dopamine (DA) through the highly sensitive electrochemical technique DPV.

The optical sensing methods are predominant in the field of point-of-care testing as they are easy and offer visual results. Colorimetric detection is the most common, and is commonly achieved using nanozymes with peroxidase or oxidase like activity.^18,19^ In this method a colored signal is produced by oxidation of chromogenic substrates such as TMB which is in turn affected by the presence of UA by reduction or scavenging of reactive oxygen species (ROS). Recent optical methods, including Surface Plasmon Resonance (SPR) and Surface Enhanced Raman Scattering (SERS) also enable label free and real-time detection in light-matter interaction at nanostructured interfaces. Examples of recognition strategies are natural enzymes such as uricase and biomimetic analogs, which are stronger and more stable. This includes aptasensors with synthetic oligonucleotides and peptide recognition elements mimicking enzyme binding. These strategies are more selective and enable a more precise detection in the biological fluids including serum, saliva and sweat.^20,21^

### 2.1. Overview of uric acid as a metabolic biomarker

Uric acid is the end product of purine metabolism in human and is an important metabolic biomarker. It is a disease biomarker. Normal levels of UA have the effect of acting as an antioxidant, whereas abnormal levels are associated with gout, kidney disease, cardiovascular disease and metabolic syndrome. HPLC and capillary electrophoresis are the conventional detection methods which have slowly been replaced by rapid and portable POCT devices.^23^ The most common currently methods of detection are electrochemical and optical. Nanomaterials, such as graphene, MXenes and nickel oxide are used in electrochemical methods to enhance the sensitivity for UA detection and to decrease interference of AA and DA. Optical sensors, especially colorimetric and fluorescent sensors are popular due to their simplicity and portability. Recent research also includes the nanozyme development like the silver nanoparticles and metal–organic frameworks (MOFs) which are more stable and less expensive than the enzyme based system of detecting the UA in biological fluids.^24,25^

### 2.2. Colorimetric sensing

Colorimetric detection is a popular technique for POCT due to its simplicity, affordability and the clear visual changes it generates. This techniques relies on the change in color of chromogenic substrates, such as 3,3′,5,5′-tetramethylbenzidine (TMB), which occurs due to UA presence.^26^ Traditionally, such systems use natural enzymes including horseradish peroxidase (HRP) and uricase, but recent advancements tend to adopt nanozymes for their improved stability, cost-effectiveness, and controllability in synthesis. Peroxidase mimicking nanozymes oxidise TMB using hydrogen peroxide (H_2_O_2_) to form a blue product. For UA, the analyte is reduced to its original, colorless form or quenches ROS, resulting in decolorization and detection of UA.^27,28^ Nanozymes including cobalt-cerium composites, silver decorated nickel oxides and MOFs have been extensively used in such systems, especially in microfluidic paper-based analytical devices. The use of oxidase like nanozymes is a recent development, using dissolved oxygen in place of H_2_O_2_, thereby enhancing stability and simplifying the test procedure. Furthermore, etching-based sensors make use of analyte induced distortion in metal nanoparticles, such as silver nanoprisms, which leads to change in localized surface plasmon resonance (LSPR) and color. For enhanced quantitative analysis, smartphone-based detection methods are also employed. Through a smartphone's camera, images of the sensing area are captured and quantified by computer-based software or smartphone apps to measure the red-green-blue (RGB) or grayscale intensity.^11,29^

### 2.3. Electrochemical sensing

Electrochemical UA sensing involves the electrical readout of electrochemical reactions occurring between UA and the sensing interface. These methods are highly attractive due to their sensitivity, cost-effectiveness and applicability for miniaturization.^30,31^ These include voltammetric, amperometric, impedimetric and potentiometric techniques. Voltammetric techniques are used to detect metabolic biomarkers. CV is usually employed to understand the reaction mechanism and the electrodes' characteristics, whereas DPV reduces background currents resulting in improved sensitivity and the ability to detect low concentrations of UA. The improved resolution of DPV facilitates discrimination of UA from common electroactive interferents such as AA and DA.^8,32–34^ Amperometric detection (current measurement at constant potential) is a common approach in commercial sensors due to its rapid response and simple measurement. Square wave voltammetry (SWV) enhances sensitivity and speed of response, and is suitable for real-time monitoring. Electrochemical impedance spectroscopy (EIS) involves measuring the charge transfer resistance to obtain information about the interface, and it can be used to assess the attachment of target molecules and for evaluating modifications made to sensors. Potentiometry measures the potential difference with little or no current and is often used in ion selective electrodes (ISEs) for non-invasive monitoring. Such systems are enhanced with new technologies such as screen-printed electrodes (SPEs) and two-dimensional nanomaterials (graphene and MXenes) associated with high conductivity and active sites for improved performance.^35,36^

### 2.4. Colorimetric *vs.* electrochemical

An example of the trade-off between sensitivity, cost and user-friendliness is using colorimetric or electrochemical approaches for detecting uric acid.^37^ As summarized in Table 2, electrochemical biosensors are known for being sensitive, fast and quantitative.^38,39^ They transform the physicochemical interaction between the analyte of interest and a recognition element into electrical signals, *e.g.*, current, potential or impedance. The use of advanced voltammetric techniques, such as Differential Pulse Voltammetry (DPV), is particularly helpful, as it reduces the background current, and increases the signal-to-noise ratio, which leads to the simultaneous detection of UA, dopamine and ascorbic acid, which are normally co-adsorbed on traditional electrodes.^40^ However, electrochemical systems frequently require special equipment (*e.g.* potentiostat) and electronic processing, and are thus more complex than optical systems. Colorimetric sensing on the other hand has garnered popularity due to its simplicity, low cost and the possibility of providing a visual output; this is in comparison to alternative methods. These approaches are based on the visible change in color of a chromogenic substrate (such as TMB) in the presence of the analyte of interest such as UA, enzymes or nanozymes. The fact that colorimetric systems can be easily transported, and analyzed with mobile devices, with the smartphone as the detector, is one of the strengths of colorimetric systems.^41^ While these methods are convenient and user-friendly, they can suffer from accuracy issues due to variations in natural light and camera sensors. When comparing the performance characteristics, electrochemical methods generally have lower limits of detection, with techniques like electrochemical impedance spectroscopy (EIS) being 1000 times more sensitive than direct measurements. However, new colorimetric sensors using nanomaterials can achieve adequate sensitivity for measuring clinically relevant urine UA concentrations in body fluids.^42,43^ The two approaches are now increasingly blending, with the use of materials like MXenes and graphene, which boost catalytic activity in both electrochemical and colorimetric sensors. In conclusion, electrochemical sensors are more suitable for highly sensitive clinical monitoring, while colorimetric approaches are more suitable for resource-poor environments, where the need is for easy, portable and user-friendly diagnostic platforms.^44,45^

**Table 2:** Comparative analytical performance of nanocomposite-based platforms for uric acid detection^a^

Method type	Sensing material	Detection mode/mechanism	Linear range (µM)	LOD (µM)	Real sample/matrix	Ref.
Colorimetric	Sawdust-deposited Ag NPs	Peroxidase-like activity/TMB oxidation	0.04–0.36	0.004	Urine, water	46
Colorimetric	2D CoBTC-MOF	Pseudo-peroxidase activity/H_2_O_2_ substrate	0.4–180	0.25	Serum, urine	33
Colorimetric	Ag@CuO/SD nanocomposite	Peroxidase-mimic catalytic oxidation	1.0–120	0.29	Serum	30
Colorimetric	Ag–NiO nanoparticles (µPAD)	Non-enzymatic image-based color change	1.0–5.0	1.51–1.77	DI water, PBS, SBF	47
Colorimetric	AgNPs@MCA	Oxidase-like activity using atmospheric O_2_	20–100	1.0	Serum	48
Electrochemical	NiO/graphene oxide nanocomposite	DPV	2.0–120	0.14	Real urine	22
Electrochemical	AuNPs@Ti_3_C_2_T_*x*_ MXene	DPV	1.0–1000	1.12	Serum, sweat	49
Electrochemical	PVP-graphene composite	LSV	4.0–1000	0.02	Diluted serum, urine	50
Electrochemical	CuO/GCE	CV	1.0–35,100	0.6	General biofluids	50
Electrochemical	3D-printed graphene/PLA	Amperometric flow analysis (BIA-MPA)	0.5–250	0.02	Spiked saliva	51
Fluorescence	TCPP-Zn_0_._1_Co_2_O_4_ nanorods	Fluorescence	0.00099–0.0052	0.000015	Human urine	52
Colorimetric	IL-Ag_2_O/activated carbon	Colorimetric	1.0–360	0.000207	Gout patient serum	46
Electrochemical	MOFdNC/AuNPs	SWV	0.05–1.0; 1.0–50.0	0.011	Milk samples	53
Electrochemical	Cu MSs/ZnO@CNTs/carbon cloth	DPV	5.0–240	0.18	Human serum	54
Electrochemical	MIP-loaded graphitic carbon nitride (GCN)	DPV	1.0–500	0.21	Human saliva	55
Electrochemical	ZnONRs-CuONSs/GCE	DPV	2.0–3000	0.50	Human serum	56
Electrochemical	CuNPs/porous carbon (CN)	DPV	0.03–175	N/A	Human serum	57
Electrochemical	AgNP-PDA-RGO/SPE	DPV	20–100; 200–1000	1.23	Artificial urine	58
Electrochemical	AuNPs/g-C_3_N_4_@CTAB/SPE	DPV	50–600	1.95	Human whole blood	59

a Phosphate-buffered saline (PBS), simulated body fluid (SBF), gold nanoparticles (AUNPs), titanium carbide MXene(Ti_3_C_2_T_*x*_), batch injection analysis with multiple-pulse amperometric detection (BIA-MPA), and polyvinylpyrrolidone (PVP).

## 3. Smart nanocomposites for metabolic biomarkers detection

The smart nanocomposites mentioned in this review can be divided into four groups: MOF-based materials, MXenes, single-atom nanozymes and carbon-based composites, all of which utilize electrochemical, colorimetric/enzyme-mimetic, fluorescence, and/or similar optical sensing mechanisms. Intelligent nanocomposites are essential for advancing technologies aimed at identifying metabolic biomarkers, especially in the identification of uric acid. These sophisticated materials, particularly those derived from MOFs, demonstrate significant porosity, facilitating improved interaction with desired molecules. Moreover, the architectures and active sites of these nanocomposites can be customized to enhance their efficiency in UA detection, rendering them powerful assets in biomedical applications. In another example, bimetals NiCo embedded into N-doped carbon (NiCo@NC) nanocomposites enable wireless visual sensing of UA by closed bipolar electrochemical devices (CBECDs) *via* physical separation for enhanced sensitivity.^53^ On the other hand, CoBTC MOFs act as peroxidase-like nanozymes that can rapidly-colorimetrically detect by controlling chromogenic reactions with the assistance of UA. MOF-based systems are highly stable, have high surface area and high sensitivity, especially in the biological media.^60^

The second category of nanozymes is composite nanozymes, comprised of transition metal oxides and carbon-based materials. Hybrid materials such as nickel oxide (NiO) nanoparticles with graphene oxide (GO) or carbon nanotubes (CNTs) selectively resolve the oxidation peaks of uric acid, dopamine and ascorbic acid. This is crucial for accurate detection in biological matrices. Additionally, porous carbon nanosheets derived from covalent-organic frameworks (COFs), such as Copper Nanoparticles/Carbon Nanosheets (CuNPs/CN) nanosheets provide good active-site dispersion leading to enhanced electrocatalytic activity and electron transfer.^61^ These materials address challenges such as poor conductivity and aggregation in single-material systems. Additions to nanocomposites include single-atom nanozymes (SANs) and noble metal decoration. SANs such as nitrogen-coordinated cobalt single atoms (A–Co–NG) enhance the catalyst efficiency of individual atoms and develop extremely sensitive sensors that can be modified to bind to UA. Likewise, gold nanoparticle-decorated MOF-derived nanoceria capitalizes on cerium oxide's oxygen storage capability and gold nanoparticles' high conductivity to form a sensitive system. These approaches are growing in popularity for decentralized, real-time monitoring of gout and other kidney diseases in wearable sensors and smartphone-based devices.^53,62^

### 3.1. Advanced nanocomposites, MOFs, MXenes and single-atom nanozymes

**Fig. 2:**
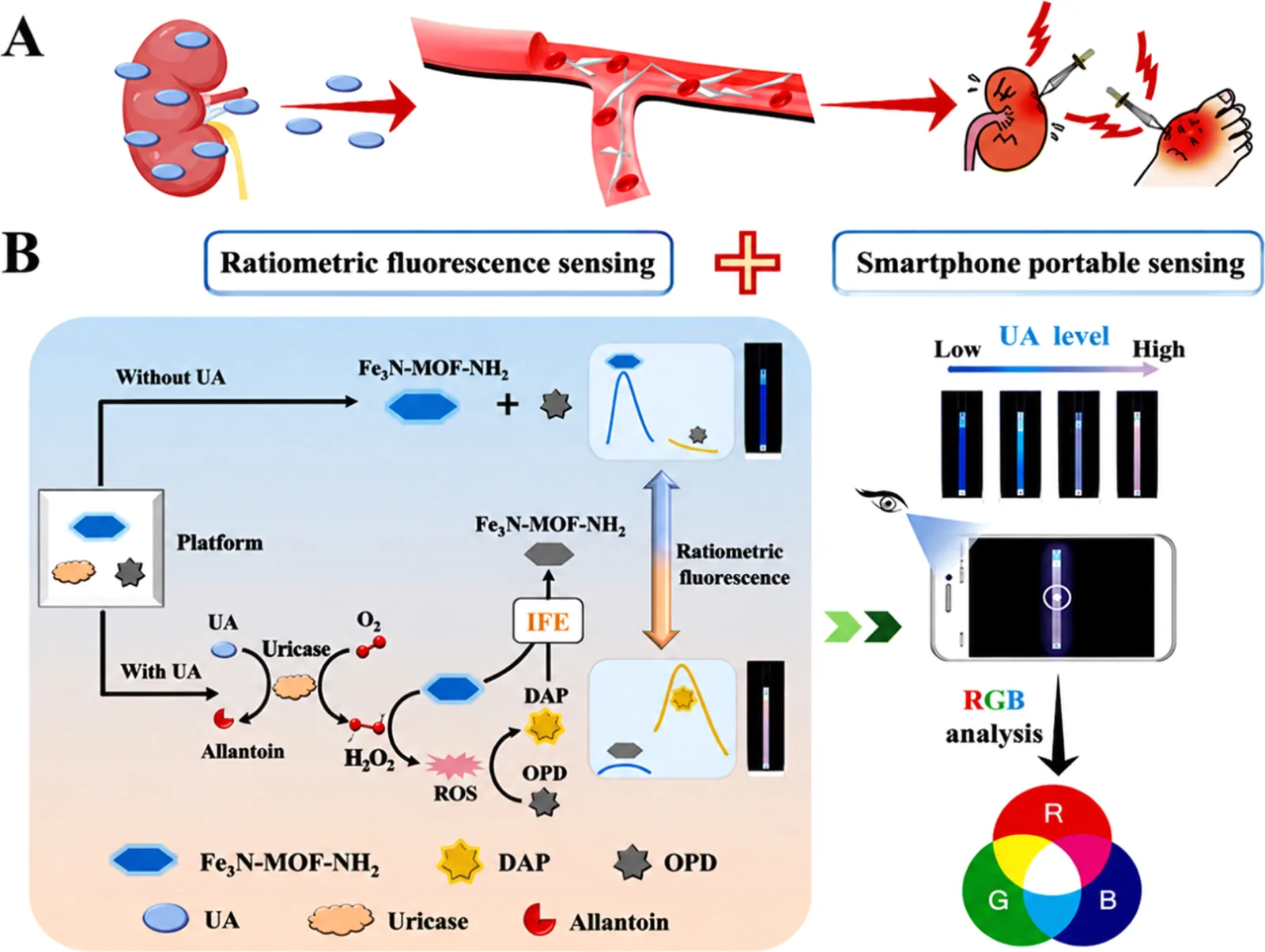
(A) The physiological pathway for uric acid formation and accumulation, and (B) representative nanocomposite based sensing strategy for the detection of uric acid to an analytical signal. This figure has been reproduced from ref. 68 with permission from Royal Society of Chemistry, copyright 2024.

New developments in smart nanocomposites to purine metabolic biomarker sensing frequently involve multifunctional high-surface-area materials that are conductive and catalyze. As briefed in Table 3, MOFs and their derivatives are more commonly used due to their tunable pore structures and coordination environments. Fig. 2 shows the physiologic background of UA formation and UA accumulation, and the relevance of this metabolic marker as a signal for metabolic sensing, and a representative nanocomposite-based sensing platform whereby recognition of the UA is translated into a measurable analytical signal, as covered in panel (A) and (B), respectively. Therefore, the two panels link together the biological significance of UA with a representative strategy for its analytical detection. As an example, CoBTC MOFs inherently possess peroxidase-like activity,^63^ which promotes the oxidation of chromogenic substrates for colorimetric analysis. More elaborate architectures, including Fe/Co/Cu trimetallic MOFs,^64^ utilize synergistic interactions between metal ions to facilitate electron transfer, and thus provide multiple detection modes, such as fluorescence, colorimetric and naked-eye detection.^65,66^ Moreover, materials based on MOF derivatives, NiCo@NC, enhance their functionality by using conductive carbon supports to entrap bimetallic particles that do not aggregate and can transfer electrons quickly. Carbon materials continue to play an important role in electrochemical sensors. Nanoparticles may be deposited onto a conductive surface, *e.g.*, graphene oxide or reduced graphene oxide.^33^ As an example, NiO/GO nanocomposites employ the oxygen functional groups on the GO to disperse NiO nanoparticles, thus augmenting the surface area and mass transport. The Multi-Walled Carbon Nanotubes (MWCNTs) and Carbon Nanofibers (CNFs) and other types of carbon materials help to create the three-dimensional conductive structures to ensure the stability of the measurement signals. Furthermore, COFs may be transformed to porous carbon nanosheets with well-dispersed sites of metals (CuNPs/CN or FeNPs/CN) to sense and selectively detect in biological samples.^67^

**Table 3:** Comparative analytical performance of advanced nanocomposite, MOF, MXene and single-atom platforms for uric acid detection

Sensing material	Material class	Detection mode	Linear range (µM)	LOD (µM)	Real sample/matrix	Ref.
A–Co–NG	Co single-atom nanozyme	Electrochemical	0.4–41,950	0.033	Human serum	78
MOFdNC/AuNPs	MOF-derived CeO_2_/Au nanocomposite	Electrochemical (SWV)	0.05–1.0; 1.0–50.0	0.011	Milk	53
Fe_3_Ni-MOF-NH_2_	Bimetallic MOF	Ratiometric fluorescence	0.2–200	0.024	Human serum	68
CoBTC-MOF	2D MOF nanozyme	Colorimetric	0.4–180	0.25	Serum, urine	33
NiCo@NC	MOF-derived bimetallic carbon	Wireless electrochromic	50–800	38.0^a^	Fetal bovine serum	75
CuNPs/CN	COF-derived Cu/porous carbon	Electrochemical (DPV)	0.03–175	0.02	Human serum	57
UOx-inorganic nanoflower diaper sensor	Enzyme-inorganic nanocomposite	Electrochemical	0–600	0.82	Urine	37
Mg@Cr/ZnO/GCE	Co-doped metal-oxide nanocomposite	Electrochemical	50–2500	2.69	Human urine	79
NiO@CNTs/GO	Metal oxide/carbon nanotube/graphene oxide nanohybrid	Electrochemical	130–1120	60	Human serum	80
Triazole-organosilane/GO	Organosiline functionalized graphene nanocomposite	Electrochemical	2.5–80	0.0657	Human urine	81
NiO/GO/GCE	Electrodeposited NiO nanoparticles and graphene oxide	Electrochemical	2.0–120	0.14	Human serum	22
ZIF-L nanosheets/SPG	MOF nanosheets	Electrochemical	0.1–900	0.04	Human urine	82
GO/WO3	Glassy carbon electrode (GCE)	Electrochemical	0.3–1245	0.306	Human urine	83
Ti–C–T_*x*_/GCE	MXene (2D Ti carbide)	Electrochemical (DPV)	0.5–4; 100–1500	0.075	Human urine	84
Fe–N–C SANzyme	Fe single-atom nanozyme	Colorimetric/smartphone	0.1–250	0.045	Human serum	85
Co–N–C SANzyme	Co single-atom nanozyme	Colorimetric	1–300	0.136	Human urine	86

a Cobalt single-atom nanozyme on N-doped graphene (A–Co–NG), MOF-derived nanoceria (MOFdNC), inner filter effect (IFE), fluorescence (FL), colorimetric (Col), and ruthenium–alanine–carbon nitride (Ru–Ala–C_3_N_4_)

**Fig. 3:**
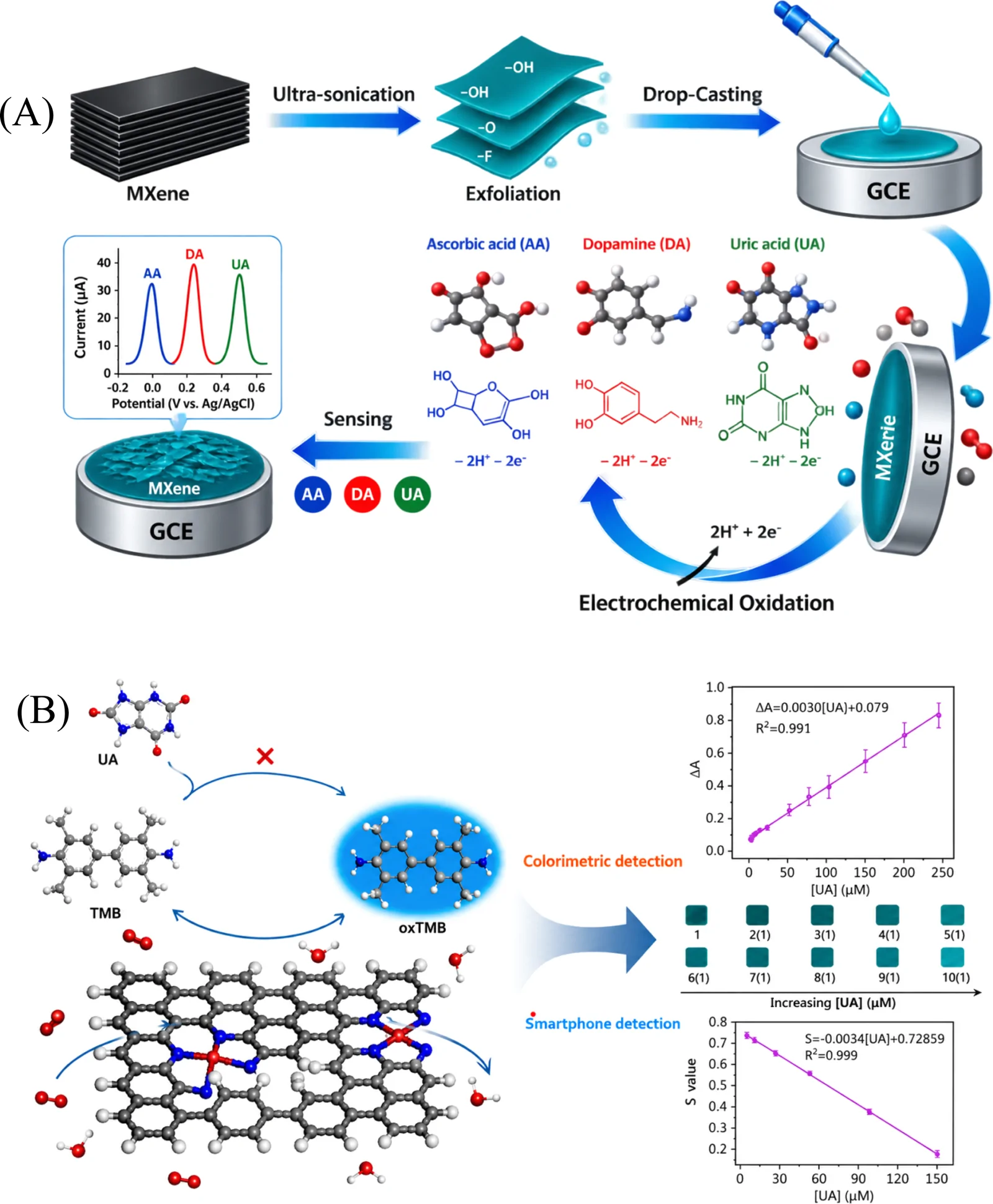
Schematic illustration of the MXene- (A) and Fe–N–C single-atom nanozyme-based platforms “this figure has been reproduced from ref. 70 with permission from ELSEVIER, copyright 2021” (B) of uric acid detection with high sensitivity “this figure has been reproduced from ref. 69 with permission from ELSEVIER, copyright 2025”.

MXenes and single-atom nanozymes are two promising material platforms for advanced UA sensing as shown in Fig. 3. The MXene Ti-based nanosheets offer a highly conductive, surface functionalized interface for fast electron transfer and electrochemical separation of UA from interfering species like ascorbic acid and dopamine. The Fe–N–C single atom nanozymes, in contrast, have the oxidase-like activity of Fe–N_4_ active sites that are highly accessible, allowing for activation of O_2_ to produce ROS, and subsequent oxidation of TMB to form oxTMB, which is blue in color. UA then reduces the colored product, giving a concentration-dependent response which can be measured colorimetrically, or using a smart phone and HSV analysis. Therefore, MXenes primarily increase the electrochemical signal resolution and electron transfer, while single atom nanozymes offer atomically defined catalytic surfaces for sensitive and portable UA detection.^69,70^

Single-atom nanozymes (SANs) are the most developed materials in the area, and they maximize the number of atoms used by anchoring single metal atoms. SANs of iron and cobalt mimic the active site of enzymes through metal–nitrogen coordination structure (M–N_*x*_) structures (M = Fe or Co, *x* = 3 or 4). This reduces the activation energy barrier and selectivity in favor of UA oxidation. Indeed, the single-atom ruthenium/carbon nitride has demonstrated high selectivity by introducing peak separation between UA and interferents (such as dopamine), a common problem. Non-enzymatic detection is also dependent on transition metal compounds. The detection of UA is highly utilized by using nickel oxide because it has a reversible pair of redox reactions that enable the oxidation of UA under alkaline conditions. Others such as cobalt oxide (Co_3_O_4_) and nickel telluride (NiTe) are also better electrocatalysts and electrode sensitive than conventional electrodes. These substances can be included in versatile systems, including wearable hydrogels, to track the UA levels in sweat and serum. These nanocomposites can form mobile and convenient diagnosis, when they are used in combination with smartphone-based readout systems.^53,71^

### 3.2. Surface functionalization for selectivity

The ability to specifically detect uric acid in the presence of other interfering molecules, such as ascorbic acid and dopamine, is important and can be achieved through surface modification. These competing species have similar oxidation potentials, which make them difficult to separate. Various functionalization methods can be employed to leverage differences in charge, size, and molecular recognition. For example, Nafion films enhance selectivity through electrostatic interactions, allowing positively charged compounds to pass through while blocking negatively charged compounds.^72,73^ On the other hand, amino-decorated MOFs provide selective binding sites that enhance selectivity and lead to preferential binding of UA molecules due to enhanced charge transfer. Selectivity is improved by the addition of recognition elements. Aptamers, synthetic nucleic acid binding molecules that selectively bind to target molecules, can be attached to the surface of nanocomposites, allowing for selective binding to UA. Similarly, molecularly imprinted polymers (MIPs) are created by polymerization in the presence of a template. This leaves cavities to selectively bind UA, even in biological samples. Such biomimetic approaches minimize interferences and improve selectivity.^74,75^

At a higher level, coordination engineering in single-atom nanozymes facilitates active site design. Structures like Co–N–C and Fe–N–C have defined coordination structures that resemble natural enzymes' active sites, facilitating UA adsorption and oxidation. Moreover, heteroatom-doping (N, S, P) of carbon matrixes alters the electronic structure and enhances molecular interactions. For example, functionalized MXene-based sensors show improved selectivity and resolution due to their surface properties. Chitosan and conductive polymers are other surface modifiers which improve the performance of sensors.^22^ They facilitate stability of catalytic nanoparticles and their dispersion and inhibit aggregation. They also help to prevent biofouling of the sensing interface by proteins and other biomolecules, a critical consideration in real biological fluids such as blood or milk. Sensitivity, stability and response time may be tuned by a careful selection and adjustment of the functional groups. In short, surface functionalization approaches, in conjunction with the design of nanocomposites, allow for accurate detection of UA. Such strategies allow the increasing demand of dependable, sensitive and practical point of care testing systems capable of operating without failure in biological conditions.^76,77^

## 4. Sensors

Synthetic methods for developing metabolic biomarsker sensors mark a substantial progress in the design of high-performance sensor materials. These strategies are noted for their ease of use, scalability, and sustainability, as they integrate all synthesis stages into one container. This reduce and simplify the requirement for multiple processing steps, thus decreasing the chances of contamination and loss of materials. Controlling the reaction conditions, such as reaction temperature, pH, time, and concentrations of active precursors, in order obtain highly efficient catalytic activity, uniform morphology and high density of active sites, which is corresponding to efficient UA sensing. The transition metal oxide nanozymes for colorimetric sensing can be prepared through one pot reduction method using suitable reducing agents. For example, nickel-doped cuprous oxide nanocauliflowers can be prepared using glucose as a green reducing agent.^35,36,87^ This method enables tuning of the Ni : Cu molar ratio (normally set to 1 : 10) for enhanced peroxidase-like activity. Similarly, porous cobalt oxide (Co_3_O_4_) can be prepared *via* one-pot combustion of cobalt nitrate with organic fuels such as glucose and glycine. The nanozymes show enzyme-like activity, excellent stability and sensitivity, with a lower detection limit (≈0.22 µM) of UA than natural enzymes.^88,89^

One-pot hydrothermal synthesis is also useful for designing fluorescent sensors for metabolic biomarker, as summarized in Table 4. Carbon quantum dots (CQDs) co-doped by heteroatoms, such as nitrogen and iron Fe/Nitrogen-Doped Carbon Dots (Fe@NCDs) can be produced by one-pot hydrothermal reactions using natural materials, such as fruit juice. This combination leads to uniform doping, high photostability and high catalytic activity. Other one-pot strategies include the preparation of porphyrin-metal oxide hybrids, such as tetrakis(4-carboxyphenyl)porphyrin (TCPP) and porous ZnCo_2_O_4_ nanorods, *via* hydrothermal methods.^90^ These composites are based on fluorescence quenching for the highly sensitive detection of UA, with detection limits down to nano-pico molar range, and demonstrate the potential for very sensitive sensors through controlled one-pot integration. One-pot processes are also widely used in the construction of electrical sensors for conducting composites.^91^ One-pot methods can be used to co-reduce noble metal nanoparticles (such as gold) and form composites with copper oxide and/or graphene supports. For instance, one-pot co-precipitation and reduction techniques are used to form uniform distributions of Au–Cu_2_O phases on reduced graphene oxide supports. This promotes electron transfer and eliminates overlapping peaks of uric acid and other biological species (*e.g.* dopamine and ascorbic acid). Similarly, photochemical one-pot reactions allow simultaneous reduction of graphene oxide and formation of catalytic phases (such as Prussian blue) to develop stable interfaces for wearable and point-of-care sensors.^37,62^

**Table 4:** Comparison of one-pot and green synthesis strategies, analytical performance, and advantages for advanced metabolic biomarker sensors

Sensing material	Synthesis technique	Precursors/green source	LOD	Linear range (µM)	Advantage/Emerging green trend	Ref.
TCPP-Zn_0.1_Co_2_O_4_ nanorods	One-pot hydrothermal	Porphyrin (TCPP) + Zn/Co salts	0.015 µM	0.001–0.005	Ultra-high sensitivity; dynamic interaction enables ultra-trace sensing	52
Ni-doped Cu_2_O nanocauliflowers	One-pot reduction	Cu/Ni salts + glucose	0.22 µM	0.4–300	Uses glucose as a benign reducing agent; superior stability to natural enzymes	101
Ag@CuO/SD (sawdust)	Green hydrothermal	Sawdust + *Punica granatum* extract	0.29 µM	1.0–120	“Waste-to-wealth”; leaf extract serves as a biogenic reducer	30
Porous Co_3_O_4_	One-step combustion	Cobalt nitrate + Glucose	0.33 µM	1.0–600	Rapid 4-min synthesis; eliminates the need for natural enzymes or H_2_O_2_	102
TiO_2_@C composite	One-step pyrolysis	MOF (MIL-125) precursor	0.09 µM	0.2–200	Overcomes poor conductivity of anatase TiO_2_*via in situ* carbon coating	101
1T/2H MoS_2_ hybrid	Single-step hydrothermal	Mo precursors	0.29 µM	0.5–100	Phase-engineered synergy; higher stability than heteroatom doping	103
Fe@NCDs (quantum dots)	One-pot hydrothermal	*Passiflora edulis* Sims extract	0.64 µM	2.0–150	Biogenic carbon source; intrinsic peroxidase-like activity	104
Au–Cu_2_O/rGO ternary	One-pot Co-precipitation	Au/Cu salts + GO	0.035 µM	0.1–1000	Streamlined ternary integration; synergistic electron transfer enhancement	100

### 4.1. One-pot synthesis methods

One-pot synthesis approaches are an integrated synthesis method in which all the precursors are introduced into a single reaction vessel to form functional nanocomposites. These methods shorten synthesis pathways, enhance reproducibility and enable control of chemical structure and electronic properties.^92^ Hydrothermal one-pot synthesis is a popular approach for preparing UA sensing materials as it allows formation of well-crystallized nanostructures under specific temperature and pressure conditions. For example, hybrid materials such as TCPP-functionalized ZnCo_2_O_4_ nanorods are hydrothermally synthesized in a one-step process that allows simultaneous formation of metal oxide hosts and integration of fluorescent porphyrin molecules. This combination leads to highly sensitive fluorescent sensors for detecting UA at low concentrations (∼15 pM). Likewise, boron and nitrogen co-doped carbon dots can be synthesized by one-pot hydrothermal carbonization, leading to highly selective optical sensors for neurotransmitters and metabolic products.^93,94^

In electrochemistry, one-pot synthesis is extensively used for preparation of ternary nanocomposites with superior conductivity and electrocatalytic properties. One-pot reactions are used to prepare Au–Cu_2_O/rGO systems, formed through simultaneous reduction and nucleation processes, which result in a uniform dispersion of active sites on graphene sheets. This structure facilitates charge transport and minimizes signal overlap. Other systems such as porphyrin SnO_2_–Au nanoparticle hybrids are also produced in a one-pot reaction, allowing for simultaneous detection of UA and epinephrine. Other single pot synthesis techniques include *in situ* polymerization and direct growth.^33,58^ Conducting polymer composites, such as porphyrin-polypyrrole hybrids, are prepared by trapping molecules into the polymer during the polymerization process, which makes them stable and conductive. Similarly, ZnO nanorods can be directly decorated with metalloporphyrin on a conductive substrate to facilitate good electron transfer and active sites. The integrated methods provide both a simpler method of sensor fabrication and improve performance and reliability.^59^

### 4.2. Advantages of one-pot synthesis

The one-pot synthesis approach has proven to be one of the most attractive methods in nanomaterial synthesis and has a number of advantages for the fabrication of UA sensors. The most apparent benefit is that the process becomes simplified as there are no multiple-step synthesis, purification and post-treatment processes. This translates into quicker fabrications, less contamination and waste, and better yield and reproducibility. Structural control is another important factor. In a single reaction, factors including precursor concentration, doping concentration, and reaction kinetics can be precisely controlled to enhance catalytic performance. For instance, bimetallic ratios in NiCu_2_O or heteroatom doping in carbon quantum dots can be precisely tuned to increase the number of active sites and boost performance. These structures enhance electron transport and promote synergistic effects between metallic and carbon components.^55,95^

One-pot strategies also lead to highly integrated nanostructures where functional components (*e.g.*, metals, oxides, and carbon supports) are homogeneously dispersed. This enhances electron transfer and resolves the oxidation peak of UA with that of interfering substances, such as dopamine. This leads to ultra-high sensitivity and very low detection limits (in the nanomolar or sub-nanomolar range). Crucially, the ease and scalability of one-pot synthesis render it suitable for practical applications. This approach enables the production of wearable, portable and point-of-care diagnostic platforms. As a result, the monitoring of UA can transition from the lab to the patient bedside and ultimately to the point of care to allow for continuous monitoring of patients with gout and other renal-related diseases.^96^

### 4.3. Emerging trends in green synthesis

Recent developments in green synthesis focus more on sustainability and biocompatibility and also ensure valorization of waste products in the production of UA sensors. Notable is the introduction of plant extracts in the form of reducing and capping agents. The polyphenols and antioxidants in biogenic agents such as *Punica granatum* leaf extracts, and *Passiflora edulis* Sims are in the formation of nanoparticles in the absence of harmful chemicals. The process is eco-friendly as well as the catalytic properties are enhanced by offering superior surface chemistry through the use of this bio-inspired approach.^97^ The other significant area of interest is the so-called waste-to-wealth strategy, according to which waste of the agricultural and industrial sectors is converted into valuable sensing materials. As an example, *Cedrus deodora* uses dust and lotus leaves as sources of carbon to produce high surface area porous nanostructures. The stability and cost-effectiveness of metal oxide catalysts is improved by the presence of eggshells and bone hydroxyapatite as durable support. They may also have superior electrocatalytic activity owing to their inherent hierarchical structures.^30,98^

Environmentally-beneficial synthesis also applies to 2D materials in safer and more environmentally friendly solvents. For example, separating of layered MoS2 in liquids using bovine serum albumin (BSA) instead of harmful chemical reagents allows safer formation of the nanosheets. Likewise, glucose is commonly employed as an eco-friendly reducing agent for the preparation of bimetallic nanostructures (such as Ni-doped Cu_2_O) with enhanced biocompatibility and stability. Overall, these new green approaches decrease environmental risks and also increase the versatility of sensors. They facilitate the creation of cost-effective, scalable, and biocompatible diagnostic systems for wearable devices and real-time monitoring in bodily fluids like sweat, urine, and serum.^99,100^

## 5. Recent developments in one-pot colorimetric sensors for purine metabolic biomarker

The recent progress in one-pot colorimetric sensors for uric acid make a major contribution to the area of analytical diagnosis because they replace labile natural enzymes with stable nanozymes that are synthesized by simple, green and eco-friendly methods. These sensors are highly desirable for point-of-care testing due to their quick and easily interpreted visual read-outs. One-pot approaches including hydrothermal synthesis, salt-melting reactions, photochemical reduction and co-precipitation *in situ* allow the formation of highly uniform nanocomposites with a single-step reaction, eliminating contamination, improving product yield, and facilitating the uniform distribution of catalytic active sites.^20,79^

The research focus is now on green and biogenic approaches in one-pot synthesis. Plant extracts, agricultural byproducts and waste products are being employed as reducing agents and templates. An illustrative example is the synthesis of silver-copper oxide nanocomposite on sawdust (Ag@CuO/SD), achieved through a single step hydrothermal process using *Punica granatum* leaf extract as reducing agent and *Cedrus deodara* sawdust as deagglomerating agent. The combination of the plasmonic and catalytic properties of silver and CuO, respectively, leads to a highly sensitive sensor with a detection limit of 0.29 µM towards UA.^105^ Such a waste to wealth strategy not only enhances the performance but also promotes green chemistry. A key development is the use of ionic liquid (IL) in a one-pot synthesis for enhanced stability, conductivity and catalyst activity.^106^ Ionic liquids act as solvent and stabilizer to prevent aggregation of nanoparticles and to allow them to be dispersed on carbon support surfaces. For instance, IL functionalized Ag_2_O/activated carbon (IL-Ag_2_O/AC) and IL/Ag-ZnO@AC nanocomposites demonstrate excellent peroxidase catalytic activity. These facilitate the Oxidized 3,3′,5,5′-tetramethylbenzidine (oxTMB) of 3,3′,5,5′-tetramethylbenzidine (TMB) to a blue-green color (oxTMB) and the reduction of the oxTMB to blue TMB by uric acid during a redox reaction. This makes it possible to quantify UA in physiological concentrations (0.5–180 µM) including both normal and pathological levels using a colorimetric reaction.^107–110^

Recent one-pot colorimetric designs also include the use of metal–organic frameworks (MOFs) and single-atom nanozymes (SANs). Prepared by a one-step reaction, two-dimensional CoBTC MOFs have intrinsic enzyme-like activity and stability (better than natural uricase) and can be applied in continuous detection. Likewise, Fe–N–C single-atom nanozymes are atomically efficient (near 100%) and can isolate Fe sites similar to the enzyme active sites to selectively oxidize UA. These catalysts enhance sensitivity and catalytic capacity whilst remaining simple.^111^ Multi-modal sensors, such as Fe-carbon dots (Fe-CDs) with single-atom molybdenum nanoflower (Fe-CDs@MoSA-NFs) also have both colorimetric and fluorescent readouts, enabling self-calibration and eliminating false signals.^82^ The combination of one-pot colorimetric sensors and digital technology through a smartphone is the next frontier of applications. The colour changes with imaging using the smartphone camera and digital analysis of the RGB or grayscale intensity using smart phone apps can accurately quantitate the UA concentration. This is a laboratory-on-a-chip (LOAC) technology and provides real-time, low-cost and user-friendly analysis in a resource-limited setting. Overall, these advances suggest that one-pot colorimetric sensors are emerging as a powerful technology for point-of-care diagnostic monitoring of gout, hyperuricemia and renal failure.^112,113^

### 5.1. Nanoparticle-based sensors

Nanoparticle based sensors have significantly revolutionized medical testing by replacing fragile enzymes with stable and efficient nanozymes. These platforms can overcome many limitations of traditional enzyme based sensors methods, including high cost, short shelf life, limited stability, and sensitivity to environmental condition.^26^ The large surface-area-to-volume ratio and catalytic activity of metal and metal-oxide nanoparticles makes them ideal for the detection of metabolites such as uric acid, glucose and dopamine used in diagnosing metabolic conditions like gout and kidney disease.^114^ Visual detection is important in colorimetric nanoparticle sensors. There is a clear emphasis on the use of environmentally-safe synthesis methods that use natural extracts and wastes. For example, Ag@CuO/sawdust nanocomposites synthesized with *Punica granatum* extract are peroxidase mimics which convert TMB to produce a blue product. UA then reduces this back to colorless, enabling simple visual detection (detection limit 0.29 µM). These types of systems are usually coupled with smartphone image processing to allow quantitative analysis of the color change for point-of-care diagnostics.^115,116^

Nanoparticle-based electrochemical sensors have also been rapidly developed, particularly with the use of carbon nanomaterials such as graphene oxide, reduced graphene oxide and CNTs.^117^ These materials enhance electron transport and resolve overlapping UA, dopamine and ascorbic acid oxidation peaks. For instance, Cu microsphere/ZnO@CNTs/carbon cloth sensors are flexible, sensitive and have a short response time (0.1 s) for real-time measurements. Metal nanoceria (CeO_2_) derived from MOFs and combined with gold nanoparticles boost catalytic activity in biological fluids (milk and serum). Ratiometric fluorescent MOFs (*e.g.* Fe_3_Ni-MOF-NH_2_) have two fluorescence peaks for self-calibrating. They have multiple output signals (electrochemical and optical) which improve sensitivity and selectivity. The new type of nanoparticle sensors is single-atom nanozymes. These sensors are single atoms anchored on carbon and inorganic supports with nearly 100% atom efficiency. An example is nitrogen-coordinated cobalt (Co–N–C) and iron SANs that resemble enzyme active sites and lower the energy barrier for UA oxidation. They are nanomolar sensitive and selective. Also, hybrid flexible electronics enable development of wearable sensors including hydrogel patches and even smart diapers for detecting biomarkers in sweat and urine. New electrochemiluminescence (ECL) and surface plasmon resonance (SPR) sensors also enhance the sensitivity to the picomolar level. Selectivity in clinical samples is enhanced by porphyrin-based hydrogen-bonded organic frameworks, MXene-gold nanocluster composites and machine learning signal processing. Overall, nanoparticle sensors are driving the path from bench to bedside with high sensitivity, selectivity and portability.^118,119^

### 5.2. Carbon nanomaterial-based sensors

Carbon nanomaterials are of great significance in sensor technologies due to their conductivity, stability and surface area. Such nanomaterials comprise zero-dimensional carbon dots, one-dimensional CNTs and two-dimensional graphene. These nanomaterials are commonly utilized for UA and dopamine sensors due to their stability, sensitivity, and enzyme-free environment.^120^ CNTs, particularly MWCNTs, play an important role in electrochemical sensors. Their length-to-diameter ratio and conductivity ensure fast electron transfer and catalysis. Carboxylic acid-functionalized CNTs increase their dispersion and provide negative charges to bind the positively charged dopamine molecules. Composites such as Cu microsphere/ZnO@CNTs/carbon cloth have a significant synergistic effect by combining the catalytic activity of metal oxides and conductivity of CNTs for a rapid response (0.1 s) and sensitivity. CNT electrospun nanofibers also boost diffusion and porosity for better sensitivity in biological samples.^81,121^Fig. 3 illustrates a diagram of the procedure for developing a carbon paste (CP)-based fabric electrode and its electrochemical properties for uric acid detection.

**Fig. 4:**
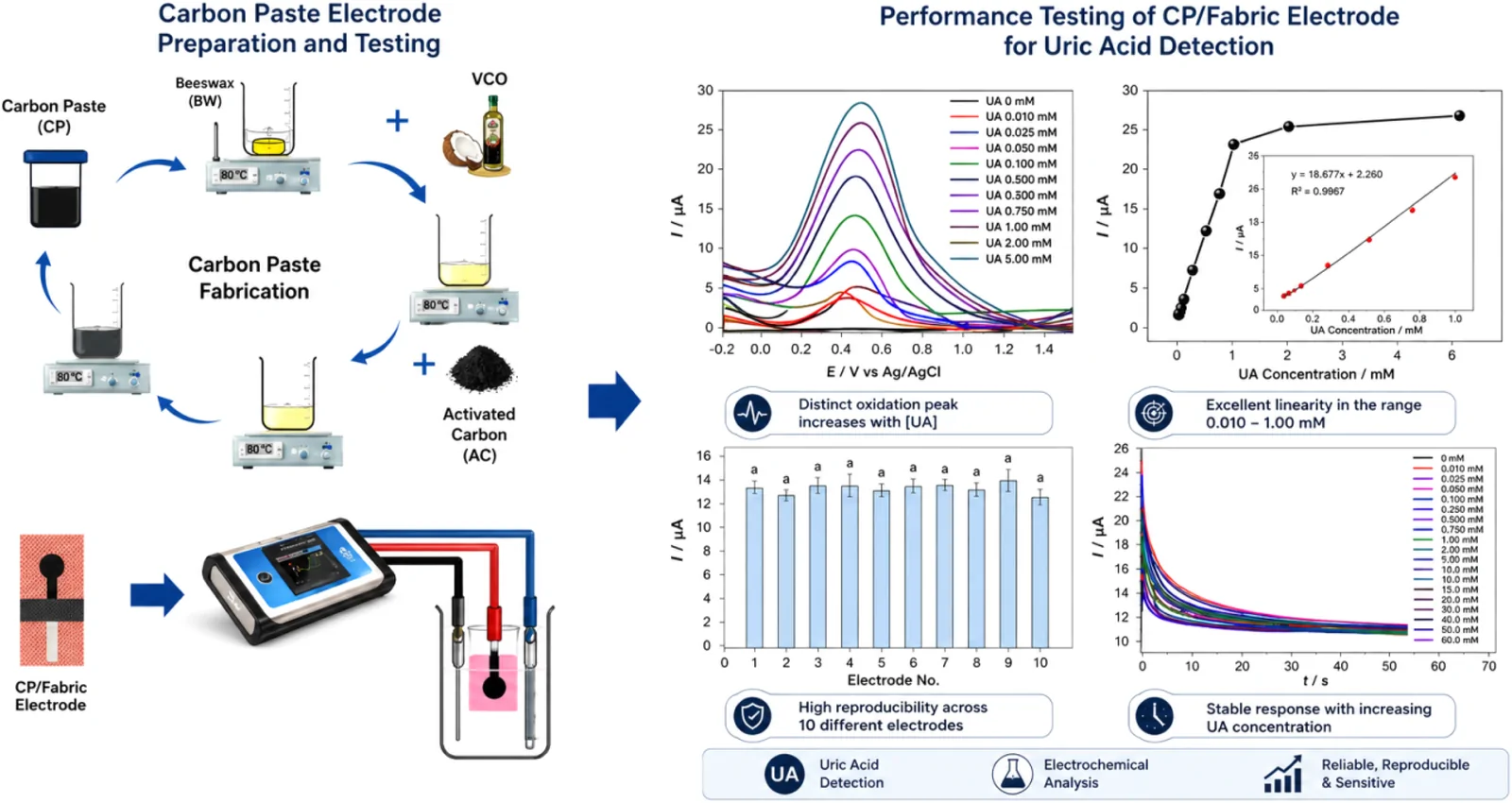
Schematic illustration of the fabrication of a carbon paste (CP)-based fabric electrode and its electrochemical performance for uric acid detection. This figure has been reproduced from ref. 121 with permission from ELSEVIER, copyright 2025.

Graphene and its derivatives, such as GO and rGO, are also important for UA detection. rGO has good electrical conductivity and charge mobility, enabling simultaneous sensing of overlapping peaks of UA,^122,123^ dopamine and ascorbic acid. Fe-Ni-MOF/rGO composites have large linear ranges (0.005–5000 µM) due to improved charge transfer. Doped graphene improves sensitivity *via* electronic modification and reduced overpotentials. Graphene quantum dots (GQDs) are also used in dual-response sensors, which have both fluorescence and colorimetric responses to provide internal calibration and accurate measurements in complex samples. Graphitic carbon nitride (g-C_3_N_4_) is a nitrogen-rich 2D material with robust covalent bonding and electron transfer properties. It is a good platform for molecularly imprinted polymers (MIPs) to prepare synthetic receptors for UA.^124,125^ These MIP-g-C_3_N_4_ sensors are “synthetic antibodies”, enabling selective sensing in saliva and urine. In addition, g-C_3_N_4_ modified with gold nanoparticles and surfactants attracts UA through electrostatic interactions, resulting in increased signals and stability. Green carbon resources are also used in sensors. Biomass-derived carbon resources like rice straw, sawdust and agricultural byproducts are sustainable and cheap alternatives. Nitrogen-doped carbon dots (N-CDs) act as intrinsic enzymes to induce chromogenic reactions for colorimetric sensors. They are also integrated with smartphones for quantitative measurements. Lastly, stretchable carbon materials such as carbon cloth, laser-induced graphene (LIG) and carbon fibers are being used to produce wearable sensors. These are stretchable, deformable and can be used in hydrogels, patches and textiles. Sensors such as Ppy-Co-NNC modified electrodes can be used to monitor UA during exercise. So, carbon nanomaterial sensors are a versatile, low-cost and highly sensitive platform for next generation non-invasive devices^126,127^ (Fig. 4).

### 5.3. Structure–property relationships in one-pot nanocomposite sensors

The performance of one-pot nanocomposite sensors for uric acid is determined by the combined properties of different material components instead of depending on just one element. The one-pot synthesis technique provides notable advantages by integrating catalytic and conductive elements into a single interface, which improves interfacial interaction and simplifies the complexities of multi-step manufacturing procedures. This synergistic behavior is well demonstrated by the metal-oxide based composites. The incorporation of Mg in Mg@Cr/ZnO changes the surface properties and enhances the electroactive surface area, while Cr acts as a catalyst for the formation of oxygen vacancies and the transfer of electrons. This combination results in the limit of detection of approximately 2.69 µM. The antifouling and selectivity properties are also highlighted, which proves that catalytic activity is not enough and its interfacial control is necessary for the analysis of biological matrices.^30,100,128^

A similar structure–function relationship can be noticed with the addition of metal oxides to a carbon-based support. The NiO@CNTs/GO possesses the electrocatalytic activity of NiO, excellent conductivity of CNTs, and larger accessible surface area of GO. The interconnected architecture enhances the electron transfer process and separates the overlapping UA and ascorbic acid oxidation signals with a peak separation of ∼170 mV, and a reported detection limit of 60 µM. In contrast, the electrodeposited NiO/GO/GCE yields a significantly lower UA LOD of 0.14 µM in urine which demonstrates that the analytical performance varies not only with the type of constituent materials but also with the way the interface is organized and fabricated.^22,129^

Porosity is another important factor for performance improvement. Short ion-diffusion pathways and enhanced oxidation of UA are enabled by the high surface area and number of accessible active sites of two-dimensional ZIF-L nanosheets. The developed platform has a LOD of 0.04 µM and it can even discriminate between UA, dopamine, and theophylline simultaneously.^82^ Beside the two-dimensional ZIF-L nanosheets, MOF-based materials enhance the sensing properties by improving the accessibility of pores, surface chemistry and the coordination environments, rather than solely by bulk conductivity. Therefore, porous frameworks can improve sensing by increasing the accessibility of the analytes and the number of reaction sites as compared to dense interfaces of metal oxides.^128^

Selectivity is the key factor alongside enhanced surface area, electron transfer and molecular recognition. In order to incorporate recognition sites in the unmodified UA, the surface was functionalized with triazole groups on the organosilane/GO, which resulted in a reported LOD of 0.657 µM.^81^ It is also applicable to matrices like urine, tap water and lake water, emphasize on the need for the association of conductive carbon surfaces and chemically selective interfaces. This is a concept fundamentally different from the metal-oxide and porous-framework approaches where enhanced catalytic activity primarily was the result of increased activity. As a result, the parallel results obtained in comparison of these systems reveal that the same goal of low LOD and high selectivity can be achieved in several ways-electrocatalytic enhancement, accelerated charge transfer, increased active-site accessibility, or molecular recognition.^130,131^ Another trend is in the direction of sustainability and mechanical flexibility. The bio-derived CPs (like virgin coconut oil and beeswax) offer low cost, eco-friendly sensing interface and can be applied onto flexible substrates, showing reported UA working range of ∼10–1000 µM.^121^ As par the mass transfer is concerned, the vertically oriented N-doped carbon nanosheets (NCSs) grown on three-dimensional porous carbon scaffolds, taking advantages of three-level porosities and conductive pathways, are developed for improved mass transport and simultaneous diffusion of UA, dopamine, and ascorbic acid.^132^ These architectures provide evidence of a design trend moving away from designing for maximum analytical sensitivity and towards designing for sensitivity as well as flexibility, portability, sustainability and compatibility with point-of-care operation.^133,134^

Analytical performance of different sensors material is also affected by many other factors such as electrode surface area, dopant concentration, defect density, porosity, accessibility of active sites, and efficiency of charge transfer at the interfaces, which cannot be explained only by catalytic properties of the constituent materials. The apparent sensitivity and LOD can be further modified depending on the procedure employed for the electroanalysis, the applied potential, the pH of the electrolyte, its composition, and the difference in electrode preparation, material loading, *etc.* However, the differences in the picomolar and micromolar ranges between closely related nanocomposite platforms are due to both material-specific effects and variations in experimental and measurement conditions, and direct comparisons of LOD values must be done accordingly.^135^

These studies collectively demonstrate a thorough connection between structure, characteristics, and functionality in apparently unrelated nanocomposite systems. Metal doping enhances catalytic activity. Oxygen vacancies, CNTs, graphene-based materials, and conductive carbon frameworks facilitate electron transfer. The porous structures of MOFs enhance active-site accessibility and mass transport. Surface functionalization enhances molecular recognition. These material-level benefits are also extended towards wearable and decentralized sensing through 3D and flexible architectures. Thus, the highest-performing sensors in one pot are not necessarily abundant; rather, they are the ones capable of operating in an integrated interface. Despite the progress in sensors systems, it is difficult to directly compare these reported studies due to the different electrode configurations, sample matrices, concentration ranges and validation conditions. These essential attributes are vital for the future advancement of one-pot sensor designs and point-of-care uric acid assessment. Key factors encompass the careful incorporation of catalytic function, electrical conductivity, molecular identification, antifouling characteristics, and scalable production, as well as the necessity for uniform assessments in real biological specimens.^136^

## 6. Recent developments in one-pot electrochemical sensors

### 6.1 Electrode materials

Recent advancements in one-pot electrochemical sensors for uric acid have resulted in the effective creation of highly synergistic nanocomposites, improving sensitivity, selectivity, and reproducibility. Traditional detection techniques frequently rely on unreliable enzymes or complex pretreatment procedures. In comparison, one-pot methods that entail merging all reactants in one container provide economical, scalable, and convenient options for non-enzymatic detection. Recent advances in the electrode materials shift electrochemical sensing from conventional noble metal and glassy carbon electrodes to flexible, nanostructured and highly conductive hybrid electrodes. The new materials consist of carbon supports, transition metal compounds and coatings to achieve sensitivity, stability, and selectivity.

#### 6.1.1 Carbon scaffolds and 3D structures

The conductivity and surface area of carbon nanomaterials continue to be extensively utilized. Recent approaches include 3D structures which prevent active metal aggregation, and enhance the mass transport. The N-doped carbon nanosheets on porous carbon framework (3D-KSC/CCSBP) provide extensive active sites to the real-time detection of UA, dopamine and ascorbic acid.^137^ Additionally, other carbon-based materials, particularly those doped with nitrogen, enhance the transport of electrolytes and facilitate electron transfer. Reduced graphene oxide is a good electron mediator due to its functionalization. Furthermore, transparent electrodes of flexible graphene modified with copper nanowires have low resistance and excellent mechanical stability, and are superior to brittle indium tin oxide (ITO) electrodes. These carbon substrates are often modified with molecularly imprinted polymers to provide binding specificity and enhanced selectivity.^132^

#### 6.1.2 Transition metal oxides, sulfides and phosphides

Transition metal compounds are often used as economical alternatives to noble metals in catalytic applications, especially regarding electrode materials. Copper catalysts (Cu, CuO, Cu_2_O) are highly effective to oxidize UA. The structural changes, including the creation of flower-like CuO and nanosheet structures, improve the accessibility of active sites, thus boosting the efficacy in multidoping applications. In Mg@Cr/ZnO, magnesium helps to increase morphological properties and chromium forms oxygen vacancies to allow the transfer of electrons. Other than oxides, sulfides (CoNi_2_S_4_) and phosphides (NiFeP) have even better conductivity and redox capability, and are thus better suited for next-generation energy storage devices and biosensors.^79,120,138^

#### 6.1.3 Framework materials and single-atom mimics

MOFs and COFs are potential high surface area electrode materials. The ion transport is fast and the peak separation is good in ZIF-L nanosheets when dealing with urea acid, dopamine and theophylline. The large surface area is conducive to exposing analytes and decreasing limits of detection. Selectivity is enhanced with biomimetic functionalization. The PdNP@rGO electrodes used as peptide-functionalized mimics of the uricase binding site, and combining the selectivity of biological molecules with inorganic strength to provide nanomolar sensitivity do not require the delicate enzymes.^80^

#### 6.1.4 Sustainable and flexible green electrodes

Sustainability is an important consideration in electrode design. Green synthesis of electrocatalysts is accomplished using plant extracts and agricultural waste. Carbon paste electrodes (activated carbon, coconut oil and beeswax) can be printed onto textiles to create wearable sensors. Such electrodes can withstand mechanical stress and allow for non-invasive detection of UA in sweat and urine. Overall, porous carbon materials, doped metals and green materials are resulting in the development of flexible high-performance electrochemical sensors.

### 6.2. Nanocomposite modifications

In recent years, nanocomposite modifications have completely transformed electrochemical sensors, replacing fragile natural enzymes with stable, inexpensive and efficient non-enzymatic sensors. They combine with the effect of transition metal oxides, precious metal nanoparticles and carbon supports to boost charge transfer, active site density and molecular recognition. The structural and electronic density modification within these nanocomposites greatly improves their effectiveness as sensor materials for identifying uric acid, glucose, and dopamine. Recent advances increasingly focus on ternary nanocomposites and co-doping strategies to synergistically tailor surface properties, defect chemistry and charge transfer behavior for enhanced sensing performance. The Mg@Cr/ZnO composite, created *via* a one-step solid-state technique, demonstrates the advantages of material combination. Magnesium improves thermal stability and surface properties, whereas chromium aids in creating oxygen vacancies, which enhances charge transfer and increases catalytic efficacy. This results in high sensitivity (2.33 µA µM^−1^ cm^−2^) and wide linear response (50–2500 µM), relative to metal oxide sensors. Selectivity is also increased by Nafion coating, due to anti-fouling and ion exchange in biofluids.^79,139^

Similarly, Cu/Cu_2_O nanostructures on carbon dots-functionalized electrodes are highly sensitive and responsive with improved surface binding and electron transport. Furthermore, surface engineering and molecular recognition also reduce detection limits to nanomoles. Triazole-based organosilane functionalized GO creates highly UA-specific binding sites, and increases selectivity. At the bio-inorganic interface, arginine-rich peptides on PdNP@rGO nanozymes mimic uricase. This biomimetic design allows enzyme-like sensitivity and a low detection limit (∼120 nM) without the need for unstable protein enzymes. Flexible and sustainable nanocomposites are the latest developments. Green carbon materials from biomass (rice straw and sawdust) are used as scaffolds for metal nanoparticle growth in “waste-to-wealth” sensors. Printable flexible electrodes comprised of activated carbon, beeswax and virgin coconut oil can be used to create durable wearable sensor platforms for UA detection in saliva and sweat under flexing conditions. Sensor performance can also be enhanced by post-synthesis modification. Post-synthesis annealing of g-C_3_N_4_/Au hybrid nanoparticles boosts photoinduced electron transfer and Surface-Enhanced Raman Scattering (SERS) by ∼2.6 times, enabling the detection of ultra-trace levels (down to 10^−11^ M) of UA in artificial urine.^116^ Finally, nanocomposite surface modifications provide a uniform way of producing stable, sensitive and portable platforms for electrochemical sensors in clinical applications.^30^

### 6.3. Electrochemical sensing examples

New electrochemical sensors are non-enzymatic nanostructured platforms that address problems with conventional electrodes, including instability, fouling, and selectivity. Incorporation of metal oxides, carbon nanomaterials and MOFs have led to sensitive, selective, and stable detection systems for key clinical biomarkers.

#### 6.3.1. Enzymeless glucose and dopamine sensing

Copper-based nanocomposites are commonly used as glucose sensors due to their excellent catalytic properties. Cu/Cu_2_O nanostructures on carbon dots-decorated carbon cloth exhibit very high sensitivity (4510.89 µA mM^−1^ cm^−2^) and response speed (<1 s). Likewise, copper nanoparticles wrapped in electrochemically reduced graphene oxide (ERGO) have ultra-low detection limits (∼49 nM) due to enhanced conductivity and efficient distribution of active sites. GO/WO_3_ nanohybrids are highly sensitive for dopamine.^140^ Uniformly dispersed WO_3_ nanocubes on graphene oxide improve electron conduction and provide broad linear ranges (0.3–1245 µM) with excellent stability in real biological fluids like urine.^141,142^

#### 6.3.2 Niche biomedical and pharmaceutical sensors

Electrochemical sensors also find applications in biomedical and pharmaceutical fields. Perovskite-type LaNi_0.6_Fe_0.4_O_3_-CeO_2_/MWCNT composite has been reported to exhibit good sensing performance for urea, with sensitivity of 195.6 µA mM^−1^ cm^−2^ and stability.^143^ For pharmaceutical applications, Pd^2+^-doped COFs functionalised with arginine-rich peptides simulate enzyme binding sites to selectively sense norfloxacin at ∼0.031 µM. Zirconia-embedded amino carbon composites are also employed for ochratoxin A immunosensing, with better wettability and electron transfer properties.^80,121,142,144^

## 7. Applications and practical considerations

The end product of purine metabolism acts as an important clinical indicator for issues like monosodium urate crystal development in joints, hyperuricemia, kidney ailments, and neurodegenerative diseases. Although traditional laboratory diagnostic tests demonstrate significant sensitivity and specificity, they require costly equipment, skilled laboratory staff, and comprehensive sample preparation procedures. To overcome these challenges, colorimetric and electrochemical sensors have been developed to facilitate rapid, inexpensive and on-site UA detections.

### 7.1. Applications and mechanism of colorimetric sensors

Colorimetric sensors are widely employed in point-of-care testing because of their straightforwardness, affordability, and ease of interpretation. These sensors operate through chromogenic reactions, in which UA induces a color shift through redox processes. An important advancement is to replace enzymes with nanozymes. For example, metal–organic frameworks like CoBTC mimic the enzymes peroxidase, converting TMB to a blue product; UA reduces the product to a colorless solution, which can be detected visually or by spectrometry. This removes enzymes for improved stability and storage. A smartphone has also made it more convenient. Digital image analysis with RGB or grayscale enables quantitative analysis of UA similar to photometers in the lab. In addition, biorecognition engineering, such as RGPT-decorated PdNP@rGO composites, enhances selectivity by mimicking uricase binding sites and, thus, avoids the interference from dopamine and ascorbic acid in the sample.^26,53,145^

### 7.2. Sensitive and wearable electrochemical platforms

Electrochemical sensors provide enhanced sensitivity and fast response for measuring UA through changes in the redox currents at the interfaces. To overcome the poor conductivity of bare electrodes, composites such as Fe-HKUST-1/Cu_2_O, MXene/PPy and carbon-metal oxides are used to improve charge transfer and increase the number of sites for reaction.^146^ Electrochemical sensors provide low limits of detection and wide linear ranges for urine, blood and serum samples. The future of wearable biosensors in wristbands, contact lenses and diapers also offer non-invasive, real-time monitoring of UA in sweat, tears, and urine. For example, enzyme-decorated diaper-based biosensors can be used for real-time monitoring of patient metabolism and wirelessly connect to a smartphone for telemedical care.^147,148^

### 7.3. Practical considerations and matrix interference

The use of sensors in actual samples encounters numerous obstacles, especially because of proteins, fats, and salts in biological fluids, leading to issues like electrode fouling, non-specific binding, and light scattering.^149^ In electrochemical sensors, significant charge-transfer resistance leads to decreased signal accuracy, whereas colorimetric sensors are affected by turbidity and diminished clarity. Furthermore, selectivity is an essential aspect in the identification of UA, DA, and AA, since these substances demonstrate comparable oxidation traits in both types of sensors.^59^ This can be overcome by using molecularly imprinted polymers (MIPs), ratiometric sensing or selective binding interfaces. pH is also critical because UA oxidation is pH-dependent, and works best in slightly acidic to neutral buffers (pH 4–7). While nanozymes are more stable than natural enzymes, issues with long-term consistency and batch-to-batch reproducibility must be resolved to facilitate their commercialization.^150,151^

### 7.4. Point of care diagnostics

Point-of-care (POC) diagnostics focus on offering prompt biomarker assessment right at the care location, removing the requirement for laboratory examinations. These POC systems, encompassing evaluations for urinalysis, glucose, and various metabolites, enable early identification and ongoing observation of issues like monosodium urate crystals in joints, diabetes, and kidney disease.^152^ POC systems are also used in food quality control (melamine or urea in milk) and environmental analysis (heavy metals and drugs). Wearable systems like smart contact lenses, wrist monitors, and diaper embedded systems provide real-time monitoring of biofluids such as sweat, tears and urine. Mobile phone-based sensing is a significant advancement. Smartphone apps analyze color or electrical signals and communicate using Bluetooth or Near-Field Communication (NFC), allowing telehealth monitoring. To be user friendly, POC devices must meet ASSURED guidelines (affordable, sensitive, specific, user-friendly, rapid, equipment-free, and deliverable). This has led to the development of affordable nanozymes, biomass-based carbon and waste products, rather than fragile biological enzymes. But sample complexity and matrix interference often means some minimal sample preparation (often filtration or dilution).^153,154^

### 7.5. Real world challenges

The swift progress in UA sensor technology faces challenges in its clinical uses. A major issue is matrix interference, in which proteins, lipids, and various electroactive compounds in biological samples build up on the sensor surfaces. This buildup blocks the active areas of the sensors, resulting in reduced sensitivity and impaired accuracy in measurements. In the case of optical sensors, turbidity and scattering also interfere with low concentration signals. Stability is also a significant issue. Many active materials also demand simple conditions, and are not compatible with physiological conditions or wearable technology. Another problem with methods such as drop-casting or hydrothermal synthesis can lead to variation in batches of production, limiting the sensor reproducibility and scalability.^149^

## 8. Future directions and challenges

The future development of smart nanocomposite-based metabolic biomarker sensors should be considered within the same materials-fabrication-translation framework that underpins this review. One of the key emerging areas is non-insidious and wearable devices for UA monitoring. Future sensors will target sweat, saliva and tears rather than blood tests for health monitoring. Wearable sensors are being developed on flexible materials such as cotton textiles, polydimethylsiloxane (PDMS) patches and carbon cloth to form robust devices incorporating catalytic nanocomposites. For instance, carbon-based “green” electrodes with renewable pastes (activated carbon, virgin coconut oil, beeswax) offer a cost-effective means to monitor UA in real-time under mechanical deformation. Integration with smartphones is another trend. Colorimetric UA sensors based on nanozymes and smartphone apps allow quantitative analysis of UA using RGB or grayscale image analysis. This enables people to do their own tests at home without instruments. Additionally, mobile devices enable wireless connectivity to medical systems, such as Bluetooth and NFC, which enables real-time data transmission, resulting in remote patient monitoring and early detection of disease.

The path forward in the creation of clinically useful sensing systems will require an increasing sensitivity of the nanomaterials, but also bridging the gap between atomic scale materials engineering and scalable and sustainable manufacturing, as well as finally interfacing these materials with actual diagnostic devices. Functional nanocomposites and single-atom nanozymes (SANs) need to be more selective, structurally stable, antifouling, and reproducible in complex biological settings at the materials level. One-pot or green synthesis methods should be developed from the laboratory scale to become reliable and scalable, ensuring controlled composition, morphology and distribution of the active sites. From the translational perspective, these materials should be integrated into flexible, wearable, microfluidic, smartphone-driven and digitally connected, reliable and real time point-of-care testing systems. Future developments must be made based on the convergence of material engineering, sustainable synthesis and device integration, and not as independent research areas.^43,65^

### 8.1. Current analytical and materials challenges

A biological matrix poses a considerable obstacle in creating electrochemical and colorimetric sensors for uric acid. Biological samples (*e.g.*, blood, serum, urine) often contain proteins, lipids, and electroactive compounds that cause biofouling. This biofouling can hinder the active sites of electrodes, hinder the charge transfer, and reduce the rate of catalytic reactions, resulting in a loss of sensor effectiveness and accuracy. In optical sensors, turbidity and scattering affect the colorimetric responses, requiring sample pre-treatment (filtration, dilution), and hindering simplicity.^155^ Furthermore, due to similar oxidation potentials between UA with common electroactive interferents such as ascorbic acid and dopamine, sensor selectivity become vital factor. Although nanostructures sensing interfaces such as NiO@CNTs/GO and ZIFs based materials can improve the peak resolution, it is difficult to distinguish the reliable signals in complex clinical samples. Moreover, most promising advanced nanocomposite materials require precisely complex preparation methods, such as controlled doping (*e.g.* Mg@Cr/ZnO) or multi-step functionalization, which are hard to scale up and reproduce to large-scale production.

### 8.2. Biomimicry and green synthesis

Nano-sized biomimetic nanozymes will be instrumental in the replacement of the delicate natural enzymes. The recognition units of these sensors comprise the arginine-rich peptides (RGPT) that are linked to a nanostructured material like PdNP@rGO to recreate the active recognition sites of uricase. This method provides an enzyme-like selectivity and affinity, and an improved stability. We are also driving towards sustainability. The waste-to-wealth strategies use the biomass-based carbon in the wastes such as rice straw and wood sawdust to form support materials to the electrodes. These environmentally friendly materials are cost-effective and eco-friendly, which presents affordable UA sensors to the low-resource health care. The method coincides with green chemistry and a cyclical design.

### 8.3. Sensitive optical sensing for ultra-trace detection

The transduction mechanisms that can generate a highly amplified optical signal from a molecular recognition event are particularly important for sensitive optical detection of ultra-trace detection techniques such as colorimetry, fluorescence, chemiluminescence (CL), and surface-enhanced Raman scattering (SERS). Of these, the platforms based on fluorescence are especially attractive for their ease of operation and high sensitivity, and nanomaterials, such as metal–organic frameworks, can be used as fluorescent probes or signal modulators *via* photoinduced electron transfer (PET), Förster resonance energy transfer (FRET), and the inner filter effect (IFE).^156^ Fluorescence is also a very sensitive pathway, as light is only emitted as a result of the chemical reaction, with no external light source required, reducing background scattering and optical noise and allowing detection at very low concentrations. A trimetallic Fe/Co/Cu-MOF sensing platform demonstrated an ultra-low LOD for xanthine oxidase (XOD) of 0.00095 U L^−1^, with a colorimetric LOD of 0.0058 U L^−1^.^157^ Likewise, SERS provides very specific molecular fingerprinting since it focuses analytes on the surface of plasmonic nanostructures and greatly amplifies weak Raman signals. Single-atom nanozymes in which the atomically dispersed metal centers, such as Fe–N_4_ and Co–N_4_, are utilized and have enzyme-like catalytic activity, can be further integrated for improvements. The ability to combine them with porous nanostructures would pave the way to a more sensitive optical detection of biomarkers at trace/ultra-trace levels.^37,86,116^

### 8.4. Future of nanocomposite sensors

The future research efforts of next generation nanocomposite and single-atom nanozyme sensors are likely to be directed towards highly stable and selective, intelligent and interconnected sensing platforms that can operate reliably under complex physiological environments. Optimized metal–support interactions, controlled ligand coordination, and stimuli-responsive conductive polymer hydrogels can enhance catalytic stability and molecular specificity at the material level. For example, enhancing the relationships between metal atoms and their supports, as well as accurately managing ligand coordination, can be supplemented by using pH- or temperature-sensitive polyaniline matrices. Furthermore, methods like rigidification, network inter-penetration, and adding hydrophobic groups to metal–organic frameworks might be successful in hindering degradation in watery surroundings.^158^

Artificial intelligence (AI) and machine learning (ML) are increasingly acknowledged as valuable tools in extensive material screening, forecasting ideal coordination structures, uncovering new materials, and automating the evaluation of complicated sensing signals. Furthermore, pattern recognition algorithms are showing considerable analytical precision. Future advancements will probably emphasize the creation of adaptable, self-sustaining, microfluidic, and multiplexed tools aimed at ongoing biomarker observation. In addition, employing highly porous nanocomposite structures might enable the creation of closed-loop systems that provide real-time monitoring and accurately controlled drug delivery. The incorporation of technologies like the Internet of Things (IoT), smartphones, secure cloud platforms, Bluetooth, and wirelessly powered near-field communication (NFC) can improve decentralized point-of-care testing and remote health monitoring. Nonetheless, for effective clinical and commercial use, it is essential to develop standardized production methods, enhance reproducibility between batches, guarantee material recyclability, and perform comprehensive toxicity and biocompatibility evaluations.^66,158^

## 9. Conclusions

This review focuses on the rapid growth of uric acid (UA) detection based on nanocomposite platform, especially metal/metal-oxide composite materials, carbon-based materials, MOF, MXene, and single-atom nanozymes. In all these material classes, an improvement of the sensing performance is achieved by the interplay of high accessible surface area, optimized electron transfer, greater density of catalytic sites, defect/doping engineering, and molecular interactions. One-pot and green synthesis are additional areas of recent development that can help reduce the complexity and enhance the material integration and sustainability of the fabrication process. The direct comparison of reported sensors is difficult because of differences in electrode design, material loading, measurement conditions, sample matrices, and analytical approaches. Moreover, LODs can differ significantly, even between materials that have comparable compositions. Standardized performance evaluation, reproducible and scalable synthesis, antifouling behavior, long-term stability, and rigorous validation in complex biological samples should therefore be prioritized for further research. Advancement in materials, together with the creation of portable and smartphone-compatible readout systems have the potential to facilitate dependable and accessible uric acid monitoring for clinical and point-of-care uses.

## Conflicts of interest

There are no conflicts of interest to declare.

## Data Availability

No primary research results, software or code have been included and no new data were generated or analysed as part of this review.

## References

[cit1] Alkahtani S. A., Mahmoud A. M., Mahnashi M. H., AlQarni A. O., Alqahtani Y. S. A., El-Wekil M. M. (2021). Facile one pot sonochemical synthesis of layered nanostructure of ZnS NPs/rGO nanosheets for simultaneous analysis of daclatasvir and hydroxychloroquine. Microchem. J..

[cit2] Yin M., Jiao K., Dong Y., Qiu D., Han L., Li L., Hu X., Qiao X. (2026). Ratiometric fluorescence platform based on luminescence metal-organic frameworks composites for highly sensitive detection of kanamycin in food. Talanta.

[cit3] Chen D., Zheng W., Zhang Z., Yu S., Hang X., Wu H., Xiang X. W., Mu W., Jiao Y., Dong Z. (2026). Nanozymes integrated biochips toward smart detection system. Advanced Science.

[cit4] Ahmad Z., Ishfaq I., Li J., Sheoran A., Guo X., He Q., Chen Z., Liu W., Saleem F. (2025). Metallic Nanozymes: From Catalytic Mechanisms to Biomedical Therapeutics and Flexible Wearable Integration. Chem.–Asian J..

[cit5] Yang W., Lu X., Wang Y., Sun S., Liu C., Li Z. (2015). Portable and sensitive detection of protein kinase activity by using commercial personal glucose meter. Sens. Actuators, B.

[cit6] Jarnda K. V., Dai H., Ali A., Bestman P. L., Trafialek J., Roberts-Jarnda G. P., Anaman R., Kamara M. G., Wu P., Ding P. (2025). A Review on Optical Biosensors for Monitoring of Uric Acid and Blood Glucose Using Portable POCT Devices: Status, Challenges, and Future Horizons. Biosensors.

[cit7] Khattak R., Ullah R., Ullah R., Naqvi I. I., Al-Sharif M. S., Saleh D. I., Tuzen M. (2025). Kinetics and Mechanism of the Oxidation of Hexacyanoferrate(II) by Dicyanobis(2,2′-dipyridyl)iron(III) for Aqueous Dye-Sensitized Solar Cells. Int. J. Chem. Kinet..

[cit8] Ullah R., Tuzen M. (2023). Facile hydrothermal synthesis of ZnO nanoparticles on silicalite-1: Effect of ultra ZnO Nano size on desulfurization activities. Surf. Interfaces.

[cit9] Hong Q., Luo J., Wen C., Zhang J., Zhu Z., Qin S., Yuan X. (2019). Hybrid metal-graphene plasmonic sensor for multi-spectral sensing in both near- and mid-infrared ranges. Opt. Express.

[cit10] Liu L., Xia N., Yu J. (2015). A graphene oxide-based fluorescent scheme for the determination of the activity of the β-site amyloid precursor protein (BACE1) and its inhibitors. Microchim. Acta.

[cit11] Çelik H., Caf B. B., Çebi G. (2025). Smartphone-assisted biosensors in point-of-care diagnostics: integration, applications, and future challenges. Chem. Pap..

[cit12] Aslam N. M., Ullah R., Tuzen M., Ullah S., Saleh T. A. (2025). Advances in sulphite ion detection via different analytical techniques using modified metal and metal doped metal oxides nanoparticles. Inorg. Chem. Commun..

[cit13] Zhang X., Zhang Y.-C., Ma L.-X. (2016). One-pot facile fabrication of graphene-zinc oxide composite and its enhanced sensitivity for simultaneous electrochemical detection of ascorbic acid, dopamine and uric acid. Sens. Actuators, B.

[cit14] Yu L., Gao X., Liu X., Li X., Gu Z., Sun T., Gao G. (2025). Smartphone-Colorimetric Biosensor for Uric Acid through Morphological Transition of Silver Nanoplates. ACS Appl. Nano Mater..

[cit15] Xing E., Chen H., Xin X., Cui H., Dou Y., Song S. (2025). Recent advances in smart phone-based biosensors for various applications. Chemosensors.

[cit16] Cheng J., Guo J., Li X., Guo J. (2023). A smartphone-connected point-of-care photochemical biosensor for the determination of whole blood creatinine by differential optical signal readout. Biosens. Bioelectron..

[cit17] Jampa S., Wangkawee K., Tantisriyanurak S., Changpradit J., Jamieson A. M., Chaisuwan T., Luengnaruemitchai A., Wongkasemjit S. (2017). High performance and stability of copper loading on mesoporous ceria catalyst for preferential oxidation of CO in presence of excess of hydrogen. Int. J. Hydrogen Energy.

[cit18] Deng X., Yin S., Xie Z., Gao F., Jiang S., Zhou X. (2021). Synthesis of silver@platinum-cobalt nanoflower on reduced graphene oxide as an efficient catalyst for oxygen reduction reaction. Int. J. Hydrogen Energy.

[cit19] Ullah R., Tuzen M., Hazer B. (2023). Novel silver-morphine-functionalized polypropylene (AgPP-mrp) nanocomposite for the degradation of dye removal by multivariate optimization approach. Environ. Sci. Pollut. Res..

[cit20] Alizadeh N., Salimi A., Hallaj R. (2018). Mimicking peroxidase activity of Co(2)(OH)(2)CO(3)-CeO(2) nanocomposite for smartphone based detection of tumor marker using paper-based microfluidic immunodevice. Talanta.

[cit21] Pal D. B., Singh P., Mishra P. K. (2017). Composite ceria nanofiber with different copper loading using electrospinning method. J. Alloys Compd..

[cit22] Gao J., He P., Yang T., Zhou L., Wang X., Chen S., Lei H., Zhang H., Jia B., Liu J. (2019). Electrodeposited NiO/graphene oxide nanocomposite: An enhanced voltammetric sensing platform for highly sensitive detection of uric acid, dopamine and ascorbic acid. J. Electroanal. Chem..

[cit23] Muñoz M. A., Calvino J. J., Rodríguez-Izquierdo J. M., Blanco G., Arias D. C., Pérez-Omil J. A., Hernández-Garrido J. C., González-Leal J. M., Cauqui M. A., Yeste M. P. (2017). Highly stable ceria-zirconia-yttria supported Ni catalysts for syngas production by CO 2 reforming of methane. Appl. Surf. Sci..

[cit24] Wang Z., Xu L., Wang J., Wang L., Xiao J. (2017). A comparison study of adsorption of benzohydroxamic acid and amyl xanthate on smithsonite with dodecylamine as co-collector. Appl. Surf. Sci..

[cit25] Boher S., Ullah R., Tuzen M., Saleh T. A. (2023). Metal doped nanocomposites for detection of pesticides and phenolic compounds by colorimetry: trends and challenges. OpenNano.

[cit26] Liu Y., Li N., Zhang H., Wei D., Guo R. (2025). Integrating recognition peptide into a nanozyme to mimic uricase binding pocket as a multifunctional nanoplatform for highly selective uric acid recognition, sensing and degrading. Sens. Actuators, B.

[cit27] Liu Y., Jin L., Zhang H., Guo R. (2024). Direct synthesis of small-sized platinum nanoflowers loaded on carbon supports as highly efficient and stable uricase-like nanozyme. Colloids Surf., A.

[cit28] Ullah S., Li Q., Ullah R., Anwar S., Hameed M. F., Zhu M. (2023). Facile synthesis of water-soluble silver nanoclusters for the photocatalytic degradation of dyes by multivariate optimization approach. Nanoscale Adv..

[cit29] Yang D., Xu G., Yan J., Zhao L. (2025). A simple and rapid colorimetric sensor for uric acid detection based on ligand-modified silver nanoparticles with oxidase activity. Mikrochim. Acta.

[cit30] Ghayas H., Sun W., Asad M., Shah M., Khan N., Ullah R., Shahat A. A., Ibrahim M. A., Ullah M., Badshah A., Nishan U. (2024). Synthesis and Characterization of *Cedrus* deodara Sawdust-Deposited Silver and Copper Nanoparticles Using *Punica granatum* Extract for Sensing of Uric Acid. Waste Biomass Valorization.

[cit31] Gul S., Gul S., Gul H., Khitab F., Khattak R., Khan M. S., Ullah R., Ullah R., Wasil Z., Krauklis A. E. (2023). Dried leaves powder of Adiantum capillus-veneris as an efficient biosorbent for hazardous crystal violet dye from water resources. Separations.

[cit32] Ali Z., Ullah R., Tuzen M., Ullah S., Rahim A., Saleh T. A. (2023). Colorimetric sensing of heavy metals on metal doped metal oxide nanocomposites: A review. Trends Environ. Anal. Chem..

[cit33] Khaloo S. S., Vaziri M. H., Azizi F. (2025). Uricase-free and cost-effective colorimetric assay for uric acid based on CoBTC metal-organic frameworks. Sci. Rep..

[cit34] Lin J.-H., Chiang Y.-W., El-Mahdy A. F. M. (2025). Advanced Electrochemical Sensors Featuring Nitrogen-Rich Conjugated Microporous Polymers for Simultaneous Detection of Dopamine and Uric Acid. ACS Appl. Polym. Mater..

[cit35] Wang F., Shi F., Li J., Chen N., Chen C., Xu Z., Wang J. (2023). Cu microspheres decorated ZnO@CNT/Carbon cloth flexible biosensor for simultaneous determination of glucose and uric acid. Microchem. J..

[cit36] Koch M., Silina Y. E. (2024). Uric acid detection by hydrogen peroxide independent biosensors: Novel insights and applications. Microchem. J..

[cit37] Wang S., Liu W., Li W., Wang L., Luo X., Zhang G., Zhao Y. (2026). A triple-mode sensing platform for xanthine oxidase activity monitoring and inhibitor screening based on fluorescent trimetal-organic framework with efficient peroxidase-mimetic activity. Anal. Chim. Acta.

[cit38] Abamecha A., Yimer A. A., Muleta G. G., Kitte S. A. (2025). Electrochemical determination of ascorbic acid at Ag2S - CuO - ZnO ternary nanocomposite modified glassy carbon electrode. Sci. Afr..

[cit39] Ullah R., Tuzen M., Ullah S., Haroon M., Khattak R., Saleh T. A. (2023). Acidic sites enhanced ultra-deep desulfurization performance of novel NiZnO-based mixed oxides mesoporous adsorbents. Surf. Interfaces.

[cit40] He Q., Chen A., Li M., Cai J., Qiu B., Huang C., Chen Q. (2026). Gold nanoparticles/ZIF-8 MOFs as amplifier and few carbon nanotubes@β-cyclodextrin as carrier for sandwich-like electrochemical immunosensing of thyroid globulin. Talanta Open.

[cit41] Nagendran S., Palani N., Mendonce K. C., Syed Altaf R. R., Mohan A., Nithya T. G., Srinivasan M., Rajendran S., Surya P., Rajadesingu S. (2026). Metal Nanoparticle-MOF Hybrid Biosensors for Early Detection of Infectious Diseases and Antimicrobial Resistance Markers. Chempluschem.

[cit42] Khan N., Pu J., Pu C., Xu H., Gu X., Lei Z., Huang F., Nasir M. A., Ullah R. (2019). Experimental and mechanism study: Partially hydrolyzed polyacrylamide gel degradation and deplugging via ultrasonic waves and chemical agents. Ultrason. Sonochem..

[cit43] Theyagarajan K., Kim Y. J. (2024). Metal Organic Frameworks Based Wearable and Point-of-Care Electrochemical Sensors for Healthcare Monitoring. Biosensors.

[cit44] Kustov L., Vergun V., Zakharov V., Aslanov L. (2026). Metal–Organic Frameworks as Materials for Applications in Sensors for Toxicants. Crystals.

[cit45] Nagal V., Masrat S., Khan M., Alam S., Ahmad A., Alshammari M. B., Bhat K. S., Novikov S. M., Mishra P., Khosla A., Ahmad R. (2023). Highly Sensitive Electrochemical Non-Enzymatic Uric Acid Sensor Based on Cobalt Oxide Puffy Balls-like Nanostructure. Biosensors.

[cit46] Nishan U., Khan N., Muhammad N., Afridi S., Badshah A., Shah M., Asad M., Ullah R., Niamat H., Ullah R., Ali E. A., Ojha S. C. (2023). Colorimetric sensing of uric acid based on sawdust-deposited silver nanoparticles via an eco-friendly and cost-effective approach. Front. Mater..

[cit47] Govindaraj P., Alungal N., S. K. (2025). Silver conjugated nickel oxide nanoparticle dependent microfluid non-enzymatic colorimetric paper-based biosensor for uric acid detection. Biochem. Eng. J..

[cit48] Yang R., Liu Z., Chen H., Zhang X., Sun Q., El-Mesery H. S., Lu W., Dai X., Xu R. (2025). Advances in nanozyme catalysis for food safety detection: A comprehensive review on progress and challenges. Foods.

[cit49] Nguyen T. T., Zhou L., Kong J., Luo A., Hao Z., Zhang J. (2025). Recent Advances in MXene-Based Screen-Printed Electrochemical Sensors for Point-of-Care Biomarker Detections. Biosensors.

[cit50] Xun T., Zhang J., Zhang X., Wu M., Huang Y., Jiang H., Zhang X., Ding B. (2026). Functional Nanomaterial-Based Electrochemical Biosensors Enable Sensitive Detection of Disease-Related Small-Molecule Biomarkers for Diagnostics. Pharmaceuticals.

[cit51] Debnath S., Debnath T., Paul M. (2025). A Review of Graphene-Integrated Biosensors for Non-Invasive Biochemical Monitoring in Health Applications. Sensors.

[cit52] Francis S., Salim S., Rajith L. (2025). Fluorescent determination of uric acid based on porphyrin and ZnCo2O4 nanocomposite. J. Fluoresc..

[cit53] Ognjanović M., Marković M., Girman V., Nikolić V., Vranješ-Đurić S., Stanković D. M., Petković B. B. (2024). Metal–Organic Framework-Derived CeO2/Gold Nanospheres in a Highly Sensitive Electrochemical Sensor for Uric Acid Quantification in Milk. Chemosensors.

[cit54] Wang F., Shi F., Li J., Chen N., Chen C., Xu Z., Wang J. (2023). Cu microspheres decorated ZnO@ CNT/Carbon cloth flexible biosensor for simultaneous determination of glucose and uric acid. Microchem. J..

[cit55] Shahzad N., Afzal A. (2025). Molecularly imprinted nanocomposites-based synthetic antibodies for uric acid-specific non-invasive electrochemical gout sensors. Mikrochim. Acta.

[cit56] Arivazhagan M., Paramasivam S., Gnanasekaran L., Prabu S. D. S., Boonyuen S., Ravikumar A., Amalraj A., Jakmunee J. (2026). Current status and future challenges of transition-metal oxides as enzyme-mimetics for detection of clinically relevant biomarkers: a comprehensive review. Mikrochim. Acta.

[cit57] Xiong J., Yang Y., Wang L., Chen S., Du Y., Song Y. (2022). Electrochemical Sensors Based on Metal-Porous Carbon Nanozymes for Dopamine, Uric Acid and Furazolidone. Chemosensors.

[cit58] Dominguez F. A., Matabilas R. J., Montecillo J. P. R., Yago A. C. (2026). Screen-Printed Electrode Modified with Silver Nanoparticles (AgNP) Polydopamine (PDA) Reduced Graphene Oxide (RGO) Nanocomposite as a Sensor for Uric Acid. ACS Omega.

[cit59] Gallina F. C., Oliveira I. G. S., Rodrigues M. A., Basso L. F., Junior O. C., Marin B. T., Souto R. S., Pinto L. M. C., Neitzke-Abreu H. C., Lisboa T. P., Lanza M. R. V., Barros W. R. P. (2026). Development of a portable label-free electrochemical sensor modified with AuNPs/g-C(3)N(4)@CTAB for uric acid detection in complex blood samples. Mikrochim. Acta.

[cit60] Chang Z., Fu Q., Wang M., Duan D. (2025). Advances of Nanozyme-Driven Multimodal Sensing Strategies in Point-of-Care Testing. Biosensors.

[cit61] Xiong Z., Shi Z., Li M., Han J., Chen H., Ran M., Zhong S., Ma L., Ning C., Fan Y., Tang Z., Yu P. (2026). Atomically precise metal cluster enzymes for pathological tissue regeneration. BMEMat.

[cit62] Hsu C. Y., AlMohamadi H., Mohammed H., Sapaev I. B., Ballal S., Ray S., Sharma R., Chennakesavulu K., Ahmed Hamodi Z., Hussein Hamo S. (2026). The Application of Nanozymes in Colorimetric-Electrochemical Dual-Mode Biosensors for Efficient and Advanced Detection: From Mechanism to Applications. Crit. Rev. Anal. Chem..

[cit63] Liu H., Yao K., Hu M., Li S., Yang S., Zhao A. (2025). On-Chip Electrochemical Sensor Based on 3D Graphene Assembly Decorated Ultrafine RuCu Alloy Nanocatalyst for In Situ Detection of NO in Living Cells. Nanomaterials.

[cit64] Zhang Z., Li Y., Yuan Z., Wu L., Ma J., Tan W., Sun Y., Zhang G., Chai H. (2025). MOF nanozymes: active sites and sensing applications. Inorg. Chem. Front..

[cit65] Wang H., Liu C., Feng T., Zhang Y., Wu Y., Dai Z., Yi C. (2025). Enzyme-Based Electrochemical Sensing Systems for On-Site Detection: Recent Progress and Prospects. Small.

[cit66] Xu Y., Dong C., Liu X., Wei D., Chen Z., Zhou H., Li J. B., Yang Y., Tan W. (2025). Single-Atom Enzymes: From Evolutionary Insights, Precision Engineering to Breakthroughs in Biomedical Applications. Small.

[cit67] Pavadai R., Arivazhagan M., Jakmunee J., Pavadai N., Palanisamy R., Honnu G., Kityakarn S., Khumphon J., Issro C., Khamboonrueang D., Thongmee S. (2025). Highly Porous 3D Ni-MOFs as an Efficient and Enzyme-Mimic Electrochemical Sensing Platform for Glucose in Real Samples of Sweat and Saliva in Biomedical Applications. ACS Omega.

[cit68] Han J., Zhang Y., Lv X., Fan D., Dong S. (2024). A facile, low-cost bimetallic iron–nickel MOF nanozyme-propelled ratiometric fluorescent sensor for highly sensitive and selective uric acid detection and its smartphone application. Nanoscale.

[cit69] Xu D., Yang F., Ou Y., Pu Q., Chen Q., Pei H., Huang B., Wu Q., Wang Y. (2025). Highly accessible Fe–N–C single-atom nanozymes with enhanced oxidase-like activity for smartphone-assisted colorimetric detection of uric acid. Talanta.

[cit70] Murugan N., Jerome R., Preethika M., Sundaramurthy A., Sundramoorthy A. K. (2021). 2D-titanium carbide (MXene) based selective electrochemical sensor for simultaneous detection of ascorbic acid, dopamine and uric acid. J. Mater. Sci. Technol..

[cit71] Wu C., Du L., Chen W. (2025). Chemical Sensors and Biosensors Based on Metal–Organic Frameworks (MOFs). Chemosensors.

[cit72] Balkourani G., Lo Vecchio C., Baglio V., Brouzgou A., Tsiakaras P. (2026). Nafion-Treated Nickel Oxide/Graphene (Nafion-NiOx/GP) Electrocatalysts for Dopamine Detection. Catalysts.

[cit73] Ullah R., Bai P., Wu P., Liu B., Subhan F., Yan Z. (2017). Cation–anion double hydrolysis derived mesoporous mixed oxides for reactive adsorption desulfurization. Microporous Mesoporous Mater..

[cit74] Ullah R., Bai P., Wu P., Zhang Z., Zhong Z., Etim U., Subhan F., Yan Z. (2016). Comparison of the reactive adsorption desulfurization performance of Ni/ZnO–Al2O3 adsorbents prepared by different methods. Energy Fuels.

[cit75] Zhu Q., Dong H., Lu G., Qv K., Yang W., Zhang L. (2025). Wireless Bipolar Electrochromic Sensor Based on Metal–Organic Framework-Derived NiCo Bimetallic Carbon Nanocomposite for Visual Detection of Uric Acid. Chemelectrochem.

[cit76] Haider S. N.-U.-Z., Qureshi W. A., Khan S., Qaiser M. A., Ali A., Moradian J. M., Naveed A., Liu Q., Yang J., Yan Z.-F. (2026). Recent Advances in Single-Atom Nanozymes for Improved Food Safety and Nutritional Analysis. Int. J. Food Sci..

[cit77] Silva M. N. T., Rocha R. G., Richter E. M., Munoz R. A. A., Nossol E. (2023). Nickel Oxy-Hydroxy/Multi-Wall Carbon Nanotubes Film Coupled with a 3D-Printed Device as a Nonenzymatic Glucose Sensor. Biosensors.

[cit78] Xu X. Y., Lian X., Hao J. N., Zhang C., Yan B. (2017). A double-stimuli-responsive fluorescent center for monitoring of food spoilage based on dye covalently modified EuMOFs: From sensory hydrogels to logic devices. Adv. Mater..

[cit79] Marwani H. M., Saeed M., Rabbee M. F., Alahmari S. D., Khashoqji M. M., Alourfi N. M., Tayeb R. A., Madkhali O., Rahman M. M. (2026). Design of ternary novel Mg@Cr/ZnO nanocomposite based electrochemical non-enzymatic sensor for uric acid detection in biomedical application. Microchem. J..

[cit80] Ali A., Khalid A., Sajid A., Akyürekli S., Ahmed A. Y., Bayach I., Buzid A., Alharthi A. F. (2025). NiO@ CNTs wrapped graphene oxide nanohybrid towards simultaneous electrochemical detection of ascorbic acid and uric acid. Inorg. Chem. Commun..

[cit81] Singh G., Sharma S., Singh A., Pawan J., Kaur J. D., Kaur H., Mohan B., Rana S. (2024). Graphene oxide functionalized organosilanes for electrochemical detection of uric acid in urine and water. Mater. Chem. Phys..

[cit82] Beitollahi H., Tajik S., Dourandish Z., Mohammadi S. Z., Nejad F. G., Zaimbashi R. (2024). The development of a disposable voltammetric sensor based on two-dimensional leaf-like ZIF-L (Zn) modified screen-printed graphite electrode for the simultaneous analysis of uric acid, dopamine and theophylline. Inorg. Chem. Commun..

[cit83] Anbumannan V., Kumar R. R., Suresh K. (2021). Enhanced electrochemical detection of dopamine by graphene oxide/tungsten trioxide nanocomposite. Mater. Sci. Semicond. Process..

[cit84] Chen S., Yang H., Shen F., Li A., Wen S., Gao S., Bai Y., Li K., Lin Y. (2025). MXene-Based Electrochemical Sensors: Design Strategies and Application Advances for the Detection of Biological Small Molecules. Electroanalysis.

[cit85] Wang K., Hong Q., Zhu C., Xu Y., Li W., Wang Y., Chen W., Gu X., Chen X., Fang Y. (2024). Metal-ligand dual-site single-atom nanozyme mimicking urate oxidase with high substrates specificity. Nat. Commun..

[cit86] Zhang W., Huang R., Luo W., Si X., Deng D., Luo L. (2026). Application of Single-Atom Nanozymes in the Detection of Small Biomolecules: A Review. Molecules.

[cit87] Gao S., Xu X., Zheng X., Zhang Y., Zhang X. (2025). Bimetallic gold–platinum (AuPt) nanozymes: Recent advances in synthesis and applications for food safety monitoring. Foods.

[cit88] Wu J., Zhang X., Yu F., Tian M., Wang Y., Wu J., Lu W., Guo Y. (2024). Exploration of cobalt-based spinel oxide nanocatalysts MCo2O4 (M = Mn, Fe, Co, Ni, Cu, Zn) for glucose electrochemical sensing: NiCo2O4 exhibits largest Faradaic current. Chem. Eng. J..

[cit89] Yao H., Li Y., Zhan Y., Xiao B., Yan J., Liu S., Chen Z., Shu C. (2026). Novel iron–nickel bimetallic nanozyme with peroxidase-like activity for ultrasensitive uric acid detection and hyperuricaemia therapy evaluation. Nanoscale.

[cit90] Ibrahim M. R., Alneyadi S., El-Maghraby H., Wuttke S., Greish Y. (2026). Porphyrin-based zinc metal–organic framework loaded with gallic acid as a novel nanoplatform exhibiting H2O2-activated reactive oxygen species generation and cytotoxicity in breast cancer cells. RSC Adv..

[cit91] Yang H., Cao X., He Y., Zhang X., Zhang P., Wang X., Liu Y., Xu S., Fang Y., Gu L. (2025). Fe, N-CQDs triggered the fabrication of alginate encapsulated g-C(3)N(4) hydrogel for efficient photocatalytic activation of PMS and antibiotic degradation. Carbohydr. Polym..

[cit92] Mohan B., Asif M. B., Pombeiro A. J. L. (2024). Engineered Conductive Metal-organic Frameworks for Electrochemical Detection of Urinary Biomarkers. Small.

[cit93] Wei J., Wang L., Hu J., Wei W., Yang Y., Song Y., Li Y., Gao G. (2025). Development of an origami paper-based electrochemical sensor using N-doped graphene for simultaneous detection of Cd (II), Pb (II), and Hg (II) in water. Microchem. J..

[cit94] Etim U., Xu B., Ullah R., Yan Z. (2016). Effect of vanadium contamination on the framework and micropore structure of ultra stable Y-zeolite. J. Colloid Interface Sci..

[cit95] Li J., Lin H., Yan F., Cui L. (2026). A low-background, high-output electrochemiluminescence aptasensor for ultrasensitive detection of small biomolecules. Mikrochim. Acta.

[cit96] Chang Z., Fu Q., Wang M., Duan D. (2025). Advances of Nanozyme-Driven Multimodal Sensing Strategies in Point-of-Care Testing. Biosensors.

[cit97] Nasri S., Chrouda A., Ahmed Ali S. M., Mustafa B., Elamin M. B., Alhaidari L. M., Ben Halima H., Jafezic-Renault N. (2025). Green Nanoparticles for Enhanced Electrochemical Monitoring of Pharmaceutical Contaminants: Comparative Investigation Between Monometallic and Bimetallic Nanoparticles. Micromachines.

[cit98] Ullah H., Zhang X., Sun W., Ahmad I., Rabbani I., Fawy K. F., Nishan U., Badshah A. (2025). Silver-zinc oxide-doped hydroxyapatite nanocomposite: an efficient peroxidase nanozyme for the colorimetric detection of ascorbic acid. RSC Adv..

[cit99] Nishan U., Ahmed A., Muhammad N., Shah M., Asad M., Khan N., Ullah F., Ullah R., Ali E. A., Nawaz H., Badshah A. (2024). Uric acid quantification via colorimetric detection utilizing silver oxide-modified activated carbon nanoparticles functionalized with ionic liquid. RSC Adv..

[cit100] Aparna T., Sivasubramanian R., Dar M. A. (2018). One-pot synthesis of Au-Cu2O/rGO nanocomposite based electrochemical sensor for selective and simultaneous detection of dopamine and uric acid. J. Alloys Compd..

[cit101] Cheng R., Xiao Z., Tang X., Xu P., Qiu P. (2025). Nickel-doped cuprous oxide nanocauliflowers with specific peroxidase-like activity for sensitive detection of hydrogen peroxide and uric acid. Colloids Surf., B.

[cit102] Cao J., Xie C., Zeng Y., Wu Y. (2024). Enzyme-free colorimetric assay for the detection of uric acid in urine by cobalt tetroxide. Microchem. J..

[cit103] Thomas C., Sreeja S., Asha A., Reshmi R. (2025). High-performance hybrid phase 1T/2H MoS_2_ for selective and simultaneous detection of dopamine and uric acid. Electrochim. Acta.

[cit104] Liang C., Lan Y., Sun Z., Zhou L., Li Y., Liang X., Qin X. (2020). Synthesis of carbon quantum dots with iron and nitrogen from *Passiflora* edulis and their peroxidase-mimicking activity for colorimetric determination of uric acid. Microchim. Acta.

[cit105] Ghayas H., Sun W., Asad M., Shah M., Khan N., Ullah R., Shahat A. A., Ibrahim M. A., Ullah M., Badshah A. (2025). Synthesis and characterization of cedrus deodara sawdust-deposited silver and copper nanoparticles using *punica granatum* extract for sensing of uric acid. Waste Biomass Valorization.

[cit106] Daroon M., Chamjangali M. A., Ghaedi M., Sabzehmeidani M. M. (2025). Hierarchical Ti3C2Tx MXene tuned by Ag2SeO3/Bi0. 72Eu0. 28VO4 heterojunction toward enhanced photocatalytic degradation performances. J. Water Proc. Eng..

[cit107] Tariq S., Saeed U., Riaz S., Saqib A., Khurshid S., Nawaz M. H. (2024). ZnO nanoflakes decorated graphene oxide acting as an efficient platform for precise detection of uric acid via rhodamine fluorescence quenching. Mater. Today Commun..

[cit108] Subhan F., Aslam S., Yan Z., Zhen L., Ahmad A., Naeem M., Zeng J., Ullah R., Etim U. (2017). Facile functionalization of 3-D ordered KIT-6 with cuprous oxide for deep desulfurization. Chem. Eng. J..

[cit109] Etim U., Wu P., Bai P., Xing W., Ullah R., Subhan F., Yan Z. (2016). Location and surface species of fluid catalytic cracking catalyst contaminants: implications for alleviating catalyst deactivation. Energy Fuels.

[cit110] Gunasekaran S., Nasr F. A., Alotibi M. F., Rajasekar R., Srinivasan R., Al-Zharani M., Alghuraybi F. I., Perumal T. (2026). Integrating green chemistry with biomedical innovation: the role of biocompatible ionic liquids and ionic liquid nanoparticles in therapeutic applications. RSC Adv..

[cit111] Cai S., Li M., Wang S., Wen X., Liu G., Chu C. (2026). Metal–Organic Framework Nanozymes: From Rational Design and Synthesis to Biomedical Applications. Nano-Micro Lett..

[cit112] Montejo-Alvaro F., Rojas-Chávez H., Herrera-Rivera R., Mtz-Enriquez A., Cruz-Martínez H., Medina D. (2020). Investigating the stability of icosahedral Ni13–xCux (x= 1–12) bimetallic nanoclusters supported on defective graphene: Insights from first-principles calculations. Phys. E Low-dimens. Syst. Nanostruct..

[cit113] Asad M., Khan N., Khan M., Shah M., Sun W., Zhang X., Ullah R., Ali E. A., Badshah A., Nishan U. (2025). Ionic liquid-capped silver-zinc oxide@activated carbon: A Powerful nanocomposite for colorimetric uric acid detection. J. Photochem. Photobiol., A.

[cit114] Olean-Oliveira A., Teixeira M. F. S. (2018). Development of a nanocomposite chemiresistor sensor based on π-conjugated azo polymer and graphene blend for detection of dissolved oxygen. Sens. Actuators, B.

[cit115] Zhou Q., Umar A., Sodki E. M., Amine A., Xu L., Gui Y., Ibrahim A. A., Kumar R., Baskoutas S. (2018). Fabrication and characterization of highly sensitive and selective sensors based on porous NiO nanodisks. Sens. Actuators, B.

[cit116] Wang C., Cui Q., Yue T., Jiao A., Ma H., Zhang M., Zheng L., Li S., Li G., Chen M. (2022). Thermal annealing-boosted photoinduced electron transfer efficiency of g-C3N4/Au NPs hybrids for promoting SERS detection of uric acids. Vib. Spectrosc..

[cit117] Vinoth V., Subramaniyam G., Anandan S., Valdés H., Manidurai P. (2021). Non-enzymatic glucose sensor and photocurrent performance of zinc oxide quantum dots supported multi-walled carbon nanotubes. Mater. Sci. Eng. B.

[cit118] Zhang C., Fan L., Ren J., Cui M., Li N., Zhao H., Qu Y., Ji X. (2022). Facile synthesis of surface functionalized Pd(2+)@P-CDP/COFs for highly sensitive detection of norfloxacin drug based on the host-guest interaction. J. Pharm. Biomed. Anal..

[cit119] Putri Y. M. T. A., Syauqi M. I., Saputra R. J., Ernis G., Jiwanti P. K., Hartati Y. W., Kondo T., Anjani Q. K., Gunlazuardi J. (2025). Development of a boron-doped diamond/TiO2-modified SPCE electrochemical sensor for theophylline detection. Diamond Relat. Mater..

[cit120] Dong M., Hu H., Ding S., Wang C., Li L. (2021). Flexible non-enzymatic glucose biosensor based on CoNi2S4 nanosheets grown on nitrogen-doped carbon foam substrate. J. Alloys Compd..

[cit121] Sihombing Y. A., Uperianti, Edikresnha D., Anshori I., Hapidin D. A., Khairurrijal K. (2025). Screen-printed activated carbon/coconut oil/beeswax electrodes on fabrics for uric acid detection. Mater. Chem. Phys..

[cit122] Alghazzawi W., Danish E., Alnahdi H., Salam M. A. (2020). Rapid microwave-assisted hydrothermal green synthesis of rGO/NiO nanocomposite for glucose detection in diabetes. Synth. Met..

[cit123] Kim H., Kim C.-H., Kim C.-D., Luo Z., Kim T.-H. (2026). Engineering graphene oxide interfaces for electrochemical biosensing of biomolecules, cells, and organoids. Nano Converg..

[cit124] Palve Y. P., Jha N. (2020). A novel bilayer of copper nanowire and carbon nanotube electrode for highly sensitive enzyme free glucose detection. Mater. Chem. Phys..

[cit125] Dubey P. (2025). A comprehensive review on the capability of graphene quantum dots-based/involved platforms for the detection of inorganic ions. RSC Adv..

[cit126] Zhang Z., Pan P., Liu X., Yang Z., Wei J., Wei Z. (2017). 3D-copper oxide and copper oxide/few-layer graphene with screen printed nanosheet assembly for ultrasensitive non-enzymatic glucose sensing. Mater. Chem. Phys..

[cit127] Zare E., Jalilian J., Rezaei G., Vaseghi B., Mardani-Fard H. A., Taghizadeh F., Hayati R. (2025). Non-enzymatic ultrasensitive Ag/(CoOOH)1−x(NiOOH)/MIP heterostructure as a real-time optical fiber glucose sensor: An experimental and density functional theory study. Opt Laser. Technol..

[cit128] Saidi S., Mirzaei M., Torkzadeh-Mahani M., Barani M. (2025). Multivariate analysis and electrochemical detection of uric acid using a novel Fe–Ni-MOF/RGO nanosheet-based uricase biosensor on glassy carbon electrode. J. Exp. Nanosci..

[cit129] Ahmad R., Akhtar A., Nagal V., Abdullah, Sadiq M., Ahmad A., Alshammari M. B., Lee B.-I. (2026). High-performance uric acid detection using a hierarchical NiO nanostructure-based biosensor. J. Mater. Chem. B.

[cit130] Cai Z., Ye Y., Wan X., Liu J., Yang S., Xia Y., Li G., He Q. (2019). Morphology–dependent electrochemical sensing properties of iron oxide–graphene oxide nanohybrids for dopamine and uric acid. Nanomaterials.

[cit131] Zhang K., Chen X., Li Z., Wang Y., Sun S., Guo T., Zhang D., Xue Z., Zhou X., Lu X. (2018). Au-Pt bimetallic nanoparticles decorated on sulfonated nitrogen sulfur co-doped graphene for simultaneous determination of dopamine and uric acid. Talanta.

[cit132] Peng X., Xie Y., Du Y., Song Y., Chen S. (2022). Simultaneous detection of ascorbic acid, dopamine and uric acid based on vertical N-doped carbon nanosheets/three-dimensional porous carbon. J. Electroanal. Chem..

[cit133] Fernandes D. M., Costa M., Pereira C., Bachiller-Baeza B., Rodríguez-Ramos I., Guerrero-Ruiz A., Freire C. (2014). Novel electrochemical sensor based on N-doped carbon nanotubes and Fe3O4 nanoparticles: Simultaneous voltammetric determination of ascorbic acid, dopamine and uric acid. J. Colloid Interface Sci..

[cit134] Ganesan M., Ramadhass K. D., Chuang H.-C., Gopalakrishnan G. (2021). Synthesis of nitrogen-doped carbon quantum dots@ Fe2O3/multiwall carbon nanotubes ternary nanocomposite for the simultaneous electrochemical detection of 5-fluorouracil, uric acid, and xanthine. J. Mol. Liq..

[cit135] Di Pasquale G., Pollicino A. (2026). Conducting polymers for electrochemical sensing: From materials and metrology to intelligent and sustainable biointerfaces. Sensors.

[cit136] Noroozifar M., Khorasani-Motlagh M., Nadiki H. H., Hadavi M. S., Foroughi M. M. (2014). Modified fluorine-doped tin oxide electrode with inorganic ruthenium red dye-multiwalled carbon nanotubes for simultaneous determination of a dopamine, uric acid, and tryptophan. Sens. Actuators, B.

[cit137] Zhou X., He Y., Tao S., Wang J., Li F., Guo Q. (2020). Selective and simultaneous sensing of ascorbic acid, dopamine and uric acid based on nitrogen-doped mesoporous carbon. Anal. Methods.

[cit138] Fan Z., Liu B., Liu X., Li Z., Wang H., Yang S., Wang J. (2013). A flexible and disposable hybrid electrode based on Cu nanowires modified graphene transparent electrode for non-enzymatic glucose sensor. Electrochim. Acta.

[cit139] Marwani H. M., Saeed M., Rabbee M. F., Alahmari S. D., Khashoqji M. M., Alourfi N. M., Tayeb R. A., Madkhali O., Rahman M. M. (2026). Design of ternary novel Mg@ Cr/ZnO nanocomposite based electrochemical non-enzymatic sensor for uric acid detection in biomedical application. Microchem. J..

[cit140] Dahal R., Subedi K., Pathiraja G., Bastakoti B. P. (2025). Electrochemical Deposition of Cu/Cu2O Nanostructures for Enhanced CO2 Reduction Reaction. ACS Omega.

[cit141] Balasubramanian P., Velmurugan M., Chen S.-M., Hwa K.-Y. (2017). Optimized electrochemical synthesis of copper nanoparticles decorated reduced graphene oxide: Application for enzymeless determination of glucose in human blood. J. Electroanal. Chem..

[cit142] Ponnaiah S. K., Periakaruppan P., Vellaichamy B. (2018). New electrochemical sensor based on a silver-doped iron oxide nanocomposite coupled with polyaniline and its sensing application for picomolar-level detection of uric acid in human blood and urine samples. J. Phys. Chem. B.

[cit143] Sangsubun C., Chaiburi C., Maneelok S. (2026). LaNi (1−x) FexO3 perovskite catalysts prepared by high-energy ball milling for efficient air cathodes in alkaline fuel cells. RSC Adv..

[cit144] Ponnaiah S. K., Periakaruppan P. (2018). A glassy carbon electrode modified with a copper tungstate and polyaniline nanocomposite for voltammetric determination of quercetin. Microchim. Acta.

[cit145] Krid H. B., Wertani H., Hlali A., Zairi H. (2026). Graphene–Copper Hybrid Terahertz Biosensor with KNN Sensitivity Optimization for Early Skin Cancer (Melanoma) Detection. Sens. Imag..

[cit146] Ji J., Shen B., Yang F., Li S., Wang Y., Yang L., Lin H. (2026). Metal anchoring strategy: electrochemical sensor with excellent performance in gastrointestinal secretions. RSC Adv..

[cit147] Sheng L., Tan H., Zhu L., Liu K., Meng A., Li Z. (2024). In situ anchored ternary hierarchical hybrid nickel@cobaltous sulfide on poly(3,4-ethylenedioxythiophene)-reduced graphene oxide for highly efficient non-enzymatic glucose sensing. Mikrochim. Acta.

[cit148] Aburub F., Abdullah Q., Mohammad S. I., Vasudevan A., Shomurotova S., Rekha M., Agarwal A., Singh A., Sharma R., Banimadadi A. (2026). Crumpled Ti3C2Tx MXene electrodes with tunable surface chemistry for high-performance and selective electrochemical biosensing. Sci. Rep..

[cit149] Lenar N., Paczosa-Bator B. (2026). Why sensors fail in biological samples: Fouling, blocking, matrix effects and prevention solutions. Int. J. Mol. Sci..

[cit150] Ahmed J., Faisal M., Alsareii S. A., Harraz F. A. (2022). Highly sensitive and selective non-enzymatic uric acid electrochemical sensor based on novel polypyrrole-carbon black-Co3O4 nanocomposite. Adv. Compos. Hybrid Mater..

[cit151] Rodrigues E., Xu A., Sampaio P., Castro R. C., Ribeiro D. S., Santos J. L., Piloto A. M. L. (2025). Molecularly Imprinted Membranes: Dual@ MIPs@ mbr for On-Site Detection of CA 19-9. Sensors.

[cit152] Parcharoen Y., Phetkate P., Jatuworapruk K., Trif C., Pechyen C. (2026). Portable Point-of-Care Uric Acid Detection System with Cloud-Based Data Analysis and Patient Monitoring. Biosensors.

[cit153] Wan Y., Su Y., Zhu X., Liu G., Fan C. (2013). Development of electrochemical immunosensors towards point of care diagnostics. Biosens. Bioelectron..

[cit154] Zhang Y.-J., Guo L., Yu Y.-L., Wang J.-H. (2020). Photoacoustic-based miniature device with smartphone readout for point-of-care testing of uric acid. Anal. Chem..

[cit155] Chen H.-Y., Chen C. (2026). Portable Sensing Systems in Biological and Chemical Analyses: A Review of Sensor Technologies, Miniaturized Platforms, Data Processing, and Field Applications. Micromachines.

[cit156] Blachowicz T., Ehrmann G., Stepula E., Ehrmann A. (2026). Optical Biosensors—Principles of Operation and Applications. Micromachines.

[cit157] Schulman S. G. , Fluorescence and phosphorescence spectroscopy: physicochemical principles and practice, Pergamon Press, Elsevier, 2017, pp. 1–285

[cit158] Hu Z., Wang Z., Peng Y., Gao Z., Jin C., Zhuang Z., Li J.-F. (2026). Machine learning-guided design of energy-related catalysts from nanoparticles to single-atom sites. Commun. Chem..

